# Allograft transplantation for *Drosophila* tumor metastasis studies

**DOI:** 10.1242/dmm.052543

**Published:** 2025-12-29

**Authors:** Chaitali Khan, Nasser M. Rusan

**Affiliations:** Cell and Developmental Biology Center, National Heart, Lung, and Blood Institute, National Institutes of Health, Bethesda, MD 20892, USA

**Keywords:** Metastasis, *Drosophila*, Allograph transplantation, Tumor, Cancer

## Abstract

*Drosophila* has long been a powerful model for cancer research, yet the development of robust metastatic tumor models remains a challenge. Although allograft transplantation offers a promising approach, its use has been limited by technical constraints. Here, we established a reproducible serial transplantation protocol using *lgl* mutant brain tumors, enabling exponential expansion of tumor material and precise tracking of temporal tumor progression. Extending this approach to other neural stem cell (NSC)-derived tumors, we identified shared metastatic characteristics between *lgl* and *pins* mutants. Additionally, we compared tumors of different tissue origins, demonstrating that epithelial tumors, like NSC tumors, can also be serially propagated. Using these models, we showed that tumors metastasize to host organs, establish tumor colonies in the ovaries, distort the gastrointestinal tract and invade the cellular cortex of the adult brain. Overall, our study provides a systematic framework for generating metastatic tumors in adult flies from two distinct tissue origins, establishing organ-specific metastatic patterns and offering a platform for studying tumor–host interactions at secondary organ sites.

## Teaser

**Summary:** We establish reproducible *Drosophila* metastatic tumor models through serial transplantation, enabling expansion, organ-specific metastasis tracking and new opportunities to study tumor–host interactions.

## INTRODUCTION

*Drosophila* has been extensively utilized as a developmental biology and genetics model to identify novel cancer-associated genes and dissect their functional roles in tumorigenesis. These studies have significantly contributed to understanding key aspects of tumor growth, including the discovery of oncogenes and tumor suppressor genes (Brumby and Richardson, 2005; Potter et al., 2000; Rudrapatna et al., 2012; Villegas, 2019). Additionally, *Drosophila* has been widely employed to investigate mechanisms of tumor local invasion, tumor–immune system interactions and the systemic effects of tumor–host interactions (Bilder et al., 2021; Khan and Rusan, 2024; Sharpe et al., 2023). It is important to note that the lack of a closed circulatory system does limit studies involving cancer cell migration, intravasation and extravasation. However, several analogous organs with significant similarities in cellular and tissue architecture make *Drosophila* an excellent system for tackling questions addressing tumor–host interactions, tumor cell dissemination, cell invasion and cell proliferation at secondary sites (Khan and Rusan, 2024). Unfortunately, methods for studying tumor metastasis in adult *Drosophila* remain limited. A significant challenge is the low metastatic rate and insufficient development of micro- and macro-metastases in secondary organs in genetically induced tumor models, partly due to the fly's short lifespan (Campbell et al., 2019). These limitations have hindered the establishment of robust metastatic tumor models necessary for investigating tumor-host dynamics at distant sites*.*

The allograft transplantation assay was initially utilized for trans-determination studies and has been extensively used to distinguish between non-tumorous, benign and malignant tumors (Beadle and Ephrussi, 1935, 1936a,b; Gateff, 1978; Gateff and Schneiderman, 1969). A key principle of this assay is that malignant tumors can be propagated indefinitely, whereas benign tumors fail to grow upon repeated transplantation (Gateff and Schneiderman, 1974). Multiple studies have shown that mutations disrupting asymmetric cell division in larval neural stem cells (NSCs) drive aggressive tumor growth upon transplantation into the adult fly abdomen (Caussinus and Gonzalez, 2005; Gateff, 1978). Among these, *lethal giant larvae* [*lgl*; also known as *l(2)gl*], a key regulator of NSC apico-basal polarity, was the first tumor suppressor gene identified through *Drosophila* genetic studies (Gateff and Schneiderman, 1969) and one of the most extensively studied to date. Loss of *lgl* in NSCs leads to tumorigenic overgrowth of the larval brain, while transplantation of these brain fragments results in large, rapidly growing tumors in the host abdomen (Gateff, 1978; Gateff and Schneiderman, 1974). These *lgl* mutant tumors have been shown to disseminate to distant sites, including metastasize to the adult ovary (Beaucher et al., 2007a,b; Woodhouse et al., 1998). Furthermore, serial transplantation of these tumors yields increasingly aggressive growth with enhanced metastatic potential (Beaucher et al., 2007a). These findings demonstrate that the *Drosophila* allograft approach offers a powerful system for generating and studying metastatic tumors in adult hosts; however, its usage has remained limited owing to technical challenges involving primary tissue transplantation and low rate of tumor induction.

In addition to larval NSCs, neoplastic epithelial tumors derived from *lgl* mutants have also been shown to exhibit invasive behavior following allograft transplantation (Woodhouse et al., 1998). Other aggressive genotypes, such as *aPKC^−/−^ Ras^V12^* epithelial tumors, display strong local invasion during larval stages but show limited metastatic potential when transplanted into adult hosts (Kim et al., 2021). Interestingly, tumors originating from the adult gastrointestinal (GI) tract demonstrate robust growth in the host and can metastasize to secondary sites, albeit at low frequency (Campbell et al., 2019). Together, these studies highlight the differences among various tumor types in *Drosophila* allograft models, but a thorough investigation is lacking.

In this study, we utilized allograft transplantation to investigate tumorigenic and metastatic properties of larval NSCs and epithelial tissue-derived tumors. We built on a comprehensive protocol published earlier for transplanting primary tissue and serial transplantation of transplantation stage T0 tumor (Beaucher et al., 2007a; Gong et al., 2021; Rossi and Gonzalez, 2015). In our study, we established serial transplantation as a robust method for exponential expansion of T0 tumors derived from both NSCs and epithelial tissue. Specifically, we detailed tumor progression over several transplantation stages to identify a suitable stage and a time window convenient for large-scale studies. Additionally, we showed tumor progression over several days, documenting the spread tumor to distant sites. This approach enables long-term tracking and studying the temporal progression of tumors in adult hosts, adding to previous studies establishing allograft transplantation as a robust and scalable method for studying metastasis.

Using this framework, we examined NSC-derived tumors from *lgl* and *partner of inscuteable* (*pins*) mutants, revealing both similarities and key differences. Whereas *lgl* tumors contain a mixture of stem cell-like and partially differentiated neuronal cells, *pins* tumors consist predominantly of undifferentiated stem cells and exhibit high genomic instability, indicating a distinct mechanism of tumor initiation. Despite these differences, both tumor types efficiently invade secondary host tissues, seeding secondary metastases in the ovaries, and causing tissue distortion and invasion in the GI tract and brain cortex region. We further applied this method to epithelial tumors, demonstrating that, like NSC-derived tumors, epithelial tumors can be propagated using allograft transplantation. However, epithelial tumors exhibit different metastatic patterns and invade host organs with varying affinities.

Our study establishes the first standardized and efficient protocol for generating metastatic tumor that allows analysis of tumor spread to secondary organ sites in adult *Drosophila* within a practical experimental timeframe. This framework addresses key obstacles in the field by providing a reproducible system for studying metastatic progression, tumor–host interactions and organ-specific invasion dynamics. These allograft transplantation techniques offer a powerful platform for investigating metastasis *in vivo*, enabling genetic and pharmacological studies to uncover mechanisms that drive tumor dissemination and secondary site colonization.

## RESULTS

### Allograft transplantation for tumor induction using mutant NSCs

Loss of *lgl* disrupts asymmetric division of NSCs, causing symmetric self-renewal and uncontrolled NSC proliferation that drives neoplastic brain overgrowth (Gateff et al., 1974; Gateff and Schneiderman, 1969; Lee et al., 2006). Transplantation of *lgl* mutant brain lobes into adult hosts produces metastatic tumors that disseminate to distant organs, including the ovaries, highlighting their invasive and colonizing capacity (Beaucher et al., 2007a,b; Woodhouse et al., 1998). In contrast to loss of *lgl*, loss of *pins*, a key regulator of spindle orientation in NSCs, leads to failure in stem cell maintenance, resulting in overall reduced proliferation and depletion of the NSC pool (Lee et al., 2006; Siller et al., 2006). Nevertheless, like *lgl*, the *pins* mutant brain lobes form aggressive tumors upon transplantation (Caussinus and Gonzalez, 2005; Rossi and Gonzalez, 2012).

We revisited *Drosophila* allograft models to establish a robust and reproducible system for studying tumor metastasis in adult flies. To enable a comparative analysis, we focused on two distinct genotypes – *lgl* and *pins* – which exhibit opposing NSC phenotypes *in situ*. This approach allowed us to investigate how differences in NSCs affect tumor initiation and influence subsequent tumor progression and metastatic potential.

**Fig. 1.**
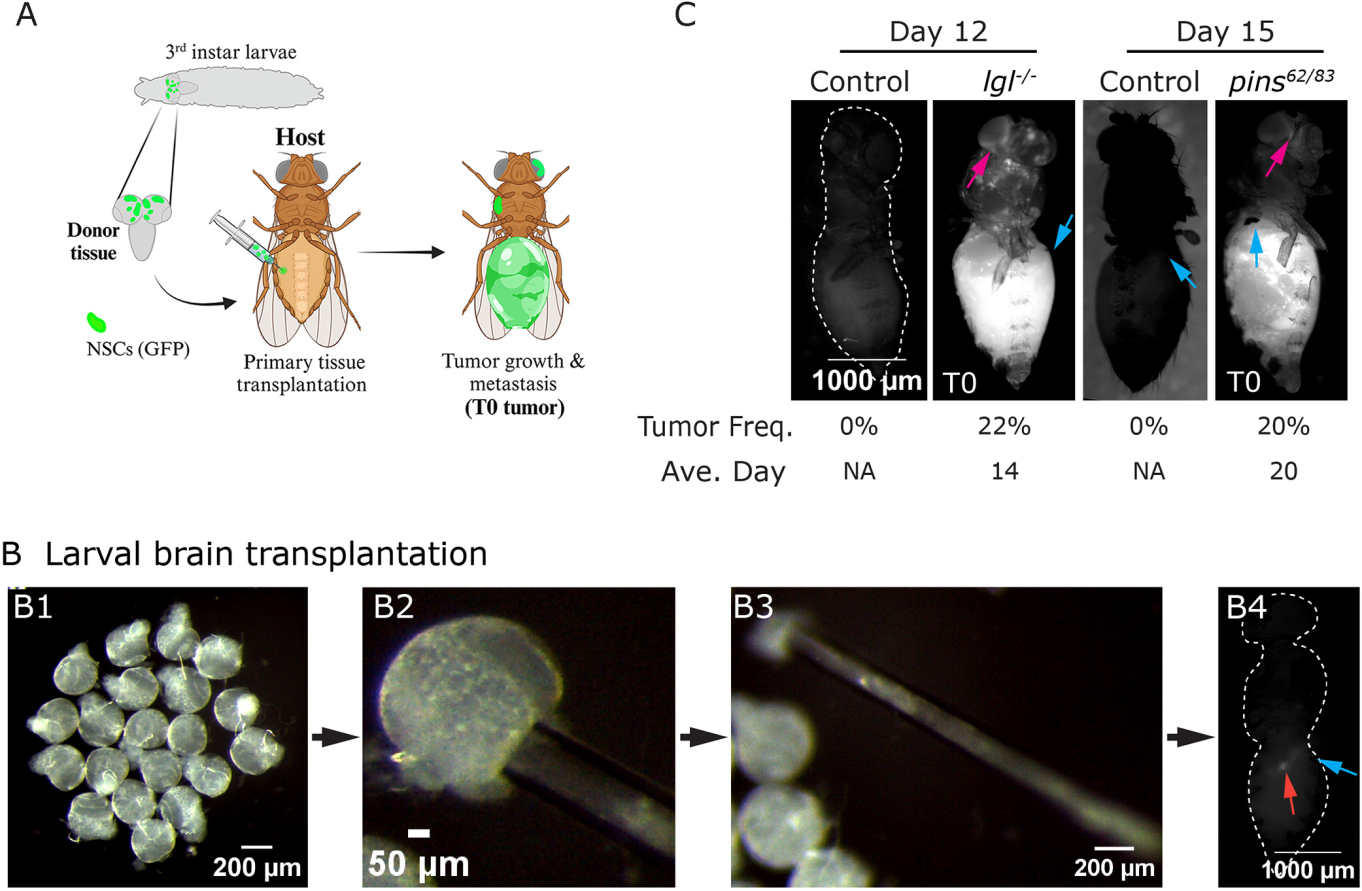
**Allograft transplantation procedure using *lgl* and *pins* mutant larval brain**. (A) Schematic illustrating the allograft transplantation procedure using the third-instar larval brain as donor tissue. NSC, neural stem cell. (B) Bright-field images illustrating key steps of the larval brain lobe transplantation procedure (Materials and Methods). B4 shows the site of the newly injected transplantation stage T0 tumor (red arrow) and the exact site of injection (blue arrow). (C) Fluorescence images of host flies taken 12 days after transplantation of brain lobes of *Frt40* control (*N*=30) and *lgl^4^-Frt40* (*N*=79), and 15 days after transplantation of *wor*-Gal4 control (*N*=15) and *pins^62/83^* (*N*=63). Magenta arrow, T0 tumor; blue arrow, site of injection. NA, not applicable. Genotypes: (C) *hsflp; Gal80-Frt40/Frt40; tub-Gal4::UAS-mCD8-RFP*, *hsflp; Gal80-Frt40/lgl^4^-Frt40; tub-Gal4::UAS-mCD8-RFP*, *wor-Gal4::UAS-mCD8-GFP* and *wor-Gal4::UAS-mCD8-GFP; pins^62^/pins^83^.*

We generated larval *Drosophila* brain lobes harboring fluorescently marked *lgl^4/4^* or *lgl^−/−^* mutant NSC clones. On average, each brain lobe contained 15 NSC clones in both mutant and control conditions (Fig. 1; Fig. S1A-C). *In situ*, each *lgl^−/−^* clone contained a single [Deadpan (Dpn)-positive] NSC and did not present with any apparent phenotype (Fig. S1A-C). Next, we transplanted *lgl* mutant or control larval brain lobes into the abdomens of 3- to 4-day-old wild-type (WT; *w^11-18^*) host female flies (Fig. 1A,B; Materials and Methods). Tumor formation was scored by assaying for fluorescence over 30 days. We found that 22% of *lgl^−/−^* transplanted brain lobes developed tumors, which took an average of 14 days, with a range of tumors appearing on Day 4 to Day 20 (Fig. 1C). We defined tumors that formed after transplantation of the primary tissue as T0, as per previous convention (Castellanos et al., 2008; Rossi and Gonzalez, 2015). After this initial growth stage, the T0 tumor grew significantly in the host's abdomen, and fluorescent cells were observed at distant sites, suggesting that *lgl^−/−^* NSC-derived tumors disseminated throughout the adult body (Fig. 1C, magenta arrows).

Next, we utilized the *pins* loss-of-function allele (*pins^62/83^*) and labeled NSCs by driving UAS-GFP with *worniu* (*wor*)-Gal4. The loss of *pins* resulted in the reduction of NSC numbers and size in larval brain lobes (Fig. 1D-F), consistent with previous findings that *pins* mutant NSCs fail to undergo self-renewal (Lee et al., 2006). Our analysis revealed that the *pins* mutant brains developed T0 tumors ∼20 days after primary tissue transplantation, with tumor formation observed in ∼20% of host flies (Fig. 1C). Despite the impaired self-renewal of *pins* mutant NSCs *in situ*, we found that their tumorigenic potential in transplantation assays was comparable to that of *lgl^−/−^* NSCs. Moreover, similar to *lgl* mutant T0 tumors, *pins* mutant T0 tumors also reached distant sites (Fig. 1C, magenta arrows).

These results were similar to previous studies on tumorigenicity of a distinct class of NSC mutant genotypes (Caussinus and Gonzalez, 2005). Our results confirm that, despite contrasting *in situ* phenotypes of NSCs, both *lgl* and *pins* show comparable capacities for tumor initiation and dissemination following allograft transplantation. This highlights a potential intrinsic property of NSC-derived tumors, irrespective of their initial proliferative nature.

### Asymmetric cell division mutants share tumorigenic potential but exhibit distinct characteristics

Although we found that T0 tumors are consistent and robust, transplantation of the primary tissue is somewhat tedious as it requires careful dissection of donor tissue, followed by gentle handling during transplantation to avoid host death. Moreover, it takes a relatively long time (2 weeks, 20% of the time for *lgl*; 20 days, 20% of the time for *pins*) to obtain T0 tumors, which poses challenges for conducting large-scale studies. To address the low frequency and long duration of T0 tumor formation, we turned to serial transplantation – a procedure in which tumors are harvested over successive generations and reimplanted into a fresh host. It was shown that increasing the proliferation rounds of *lgl* mutant tumors by culturing the tumor mass from one host to another over generations resulted in enhanced metastasis to the host ovaries (Beaucher et al., 2007b); therefore, this method seems to create more aggressive and perhaps enhanced metastatic capacity.

**Fig. 2.**
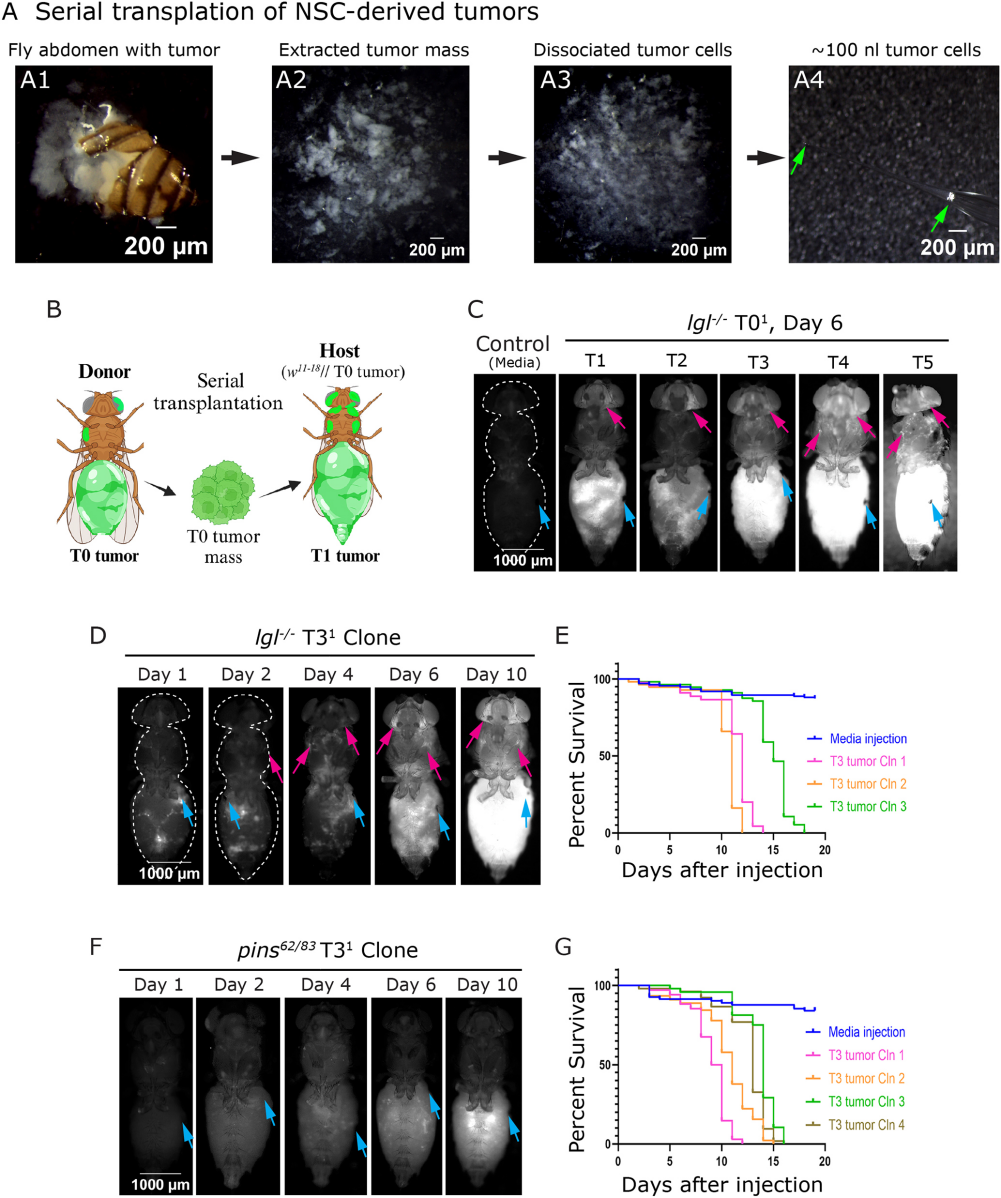
**Characterization of tumor progression of NSC-derived tumor through serial transplantation.** (A) Bright-field images illustrating key steps for performing serial transplantation of NSC-derived tumors (see Materials and Methods). (B) Schematic illustrating serial transplantation of the T0 tumor harvested from the donor and transplanted into fresh host flies (*w^11-18^*). (C) Host flies at Day 6 through different stages (T1, T2, T3, T4 and T5) after serial transplantation of the *lgl*^−/−^ tumor (clone #1). (D) Tumor progression of *lgl*^−/−^ T3 tumor at multiple days (Day 1-10) following transplantation. (E) Kaplan–Meier plot for survival fraction of host flies following transplantation of *lgl*^−/−^ T3 tumor, shown for independently generated tumor clones #1-3 (*N*=148 for control, *N*=52 for clone 1, *N*=57 for clone 2 and *N*=59 for clone 3). (F) Tumor progression of *pins^62/83^* T3 tumor photographed at multiple days (Day 1-10) following tumor transplantation (clone #1). (G) Kaplan–Meier plot for survival fraction of host flies following transplantation of *pins^62/83^* T3 tumor for independently generated tumor clones #1-4 (*N*=83 for control, *N*=45 for clone 1, *N*=48 for clone 2, *N*=52 for clone 3 and *N*=34 for clone 4). Blue arrow, site of injection; magenta arrow, tumor at distant sites; green arrow, aspirated tumor. Genotypes: (A) *w^11-18^*//*lgl^4/4^-mCD8-RFP* tumor (T0), (C) *w^11-18^*// media and *w^11-18^*//*lgl^4/4^-mCD8-RFP* tumor (T1, T2, T3, T4 and T5); (D,E) *w^11-18^*// media and *w^11-18^*//*lgl^4/4^-mCD8-RFP* tumor (T3); (F,G) *w^11-18^*// media and *w^11-18^*//*pins^62/83^-mCD8-GFP* tumor (T3).

To carry out serial transplantation, we adapted the procedure previously described in Beaucher et al. (2007a) (Fig. 2A; Materials and Methods). We selected flies bearing 10- to 15-day-old T0 tumors, which contained sufficient tumor mass to easily dissect, isolate and prepare for the next round of transplantation. A key advantage over the initial transplantation was the large size of the T0 tumor, which was sufficient material for 20-30 host injections (Fig. 2A). These secondary transplantations gave rise to flies bearing T1 tumors (Fig. 2B). Unlike the T0 tumor, the T1 tumors formed 100% of the time (versus 22% of the time for T0) and appeared, on average, 4 days after injection (versus 14 days for T0). This re-transplantation procedure was repeated over several generations, each time isolating an 8- to 10-day-old tumor mass and injecting it into a new host.

To monitor tumor progression over the course of serial transplantations, we used three independently generated T0 tumor lines (clone #T0^1^-T0^3^) and photographed the host flies at Day 6 following tumor transplantation (Fig. 2C; T0^2^ and T0^3^ lineages are not shown). We found that tumors exhibited an enhanced growth rate with progressive transplantation stages T1, T2, T3, T4 and T5 (Fig. 2C). We observed that T1 or T2 tumors grew more slowly than T3, T4 and T5 tumors (Fig. 2C). Interestingly, tumor cells spread to distant sites by Day 6, regardless of transplantation round T1-T5, as evidenced by the prominent presence of tumor cells in the head capsule (Fig. 2C, magenta arrows). From these experiments, we determined that the T3 tumors were sufficiently large and aggressive to provide material for injecting nearly 50 new hosts.

Next, we studied the course of tumor growth and metastasis in host flies. We used three independent T3 tumor clones (clone #T3^1^-T3^3^). We found that T3^1^, T3^2^ and T3^3^ all disseminated to distant sites, such as the thorax and head, as early as Day 2 (Fig. 2D; Fig. S2A,B). By Day 6, the tumor mass had filled the host abdomen and formed prominent growth at distant sites (Fig. 2D; Fig. S3A,B, magenta arrows). By Day 10, tumors occupied most of the available space in the adult flies (Fig. 2D; Fig. S2A,B). A survival assay revealed that host flies typically survived until Day 10, with a maximum survival of 14-15 days before succumbing to tumor burden (Fig. 2E). We also observed slight differences between individual clones; T3^1^ and T3^2^ were more aggressive than T3^3^ (Fig. 2D,E; Fig. S2A,B), suggesting that the tumor clones differed in their aggressiveness depending on the parental origin and subsequent changes acquired during serial transplantation. However, despite this variation, all the independent tumor clones (clone #T3^1^-T3^3^) presented a similar tumor progression pattern regarding tumor growth and dissemination to distant sites.

In summary, these studies helped establish a reproducible procedure and framework for generating metastatic tumors using *lgl* mutant NSC allograft transplantation. We were able to amplify the initial tumor burden by employing serial transplantation, enabling large-scale experiments starting at the T3 stage. Finally, we demonstrated that injected tumors provide a powerful platform for studying tumor growth and metastatic dissemination over time.

Next, we assessed the temporal progression of *pins^62/83^* mutant tumors. To enable direct comparison with *lgl* mutant tumors, we followed a parallel strategy by isolating the T3 tumor mass and transplanting it into 3- to 4-day-old adult host flies. Our results indicated that the *pins* mutant T3 tumors appeared on Day 4 (compared to Day 2 for the *lgl* mutant tumors). Additionally, *pins* tumors displayed weak GFP signal; even at Day 10, the head capsule only contained weak GFP (Fig. 2F). We repeated this experiment using four independent clones (*pins^62/83^* T3^1^-T3^4^), all of which showed only faint GFP signals at distant sites (Fig. S2C). Despite the slow tumor progression and apparent lack of distant metastasis, host survival was not significantly prolonged compared to that of *lgl* mutants; on average, 50% of host flies succumbed within 12 days of transplantation (Fig. 2G).

We hypothesized that the absence of GFP at distant sites resulted from either the inability of *pins^62/83^* tumors to migrate to these distant sites or that somehow GFP expression is lost from *pins^62/83^* tumors. To investigate these two possibilities, we isolated T3 tumor masses from the host abdomen and examined them for GFP expression. The tumor mass was easily distinguished from the host's tissue as it appeared as an independent cellular mass in the abdomen, distinct from the host's internal organs. Our analysis revealed significant loss of GFP in *pins^62/83^* tumors (Fig. S3A,A1), suggesting that the lack of detectable GFP-positive cells at distant sites was due to GFP loss in the *pins^62/83^* tumor and not an inherent inability to metastasize. Although other possible explanations exist such as epigenetic silencing of the Gal4 expression, we favor the explanation that the loss of GFP is due to the intrinsic genomic instability of *pins^62/83^* tumors. In support of this, we noticed signs of loss of heterozygosity, as evidenced by unequal inheritance of GFP copy number: one copy (Fig. S3A1′, magenta arrows) and more than two copies (Fig. S3A1′, blue arrows). We also identified multinucleated cells, which indicated that error-prone mitotic divisions could be the cause of genomic instability in the *pins^62/83^* tumor (Fig. S3A2,A3). These observation are in accordance with single-nucleotide and large-scale genetic changes in *Drosophila* malignant tumors propagated over generations by allografting (Rossi et al., 2018), but there could a possibility that *pins* tumorigenesis is driven by impaired asymmetric cell division without much contribution of genomic instability as per previous results from other NSC-derived tumors (Castellanos et al., 2008). Nevertheless, these results explain that the accelerated rate of host death from *pins^62/83^* tumors is likely to be caused by tumor spread throughout the animal (shown in Figs 5-7), but they were not detectable owing to frequent loss of GFP marker.

**Fig. 3.**
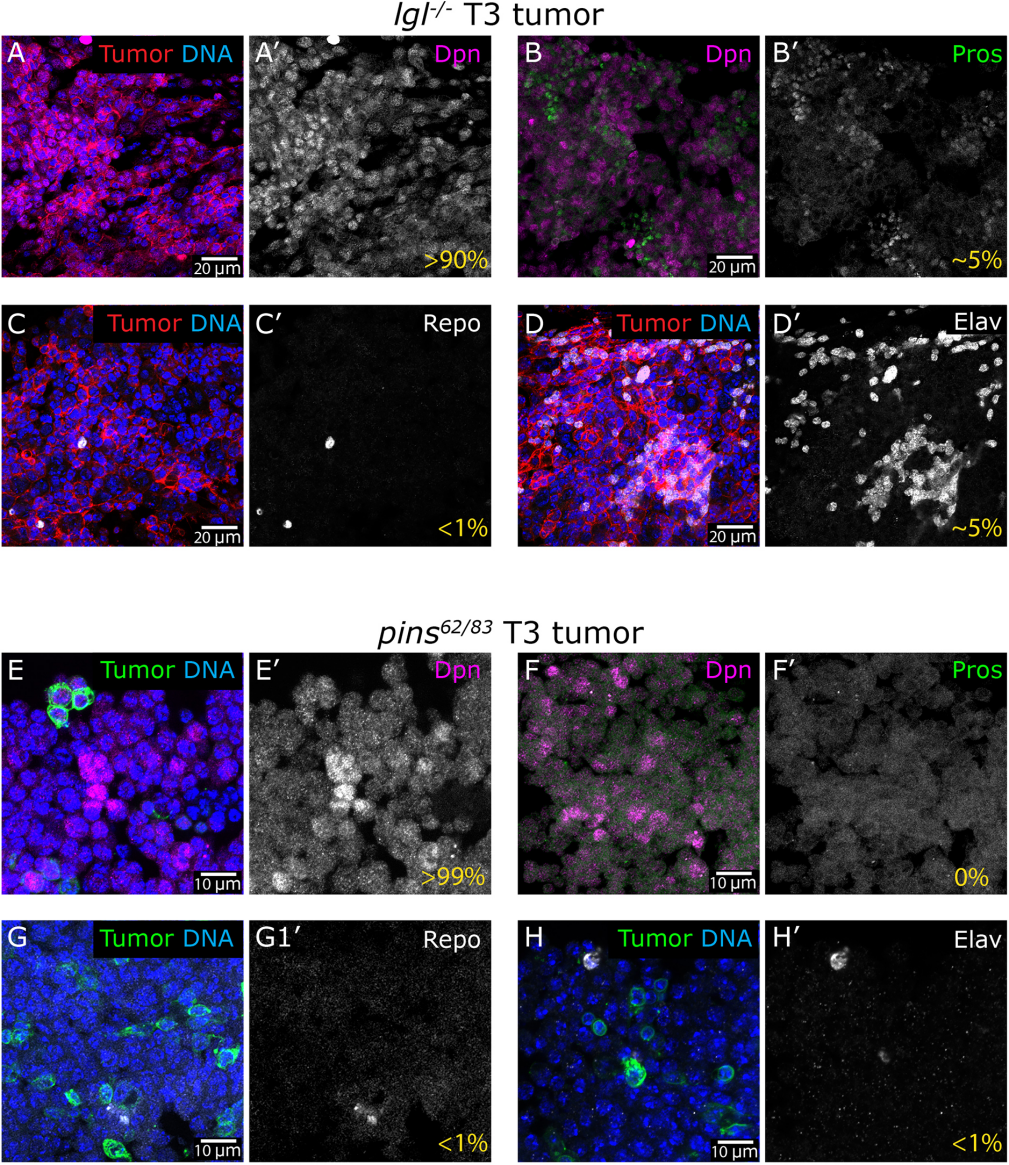
***lgl* and *pins* mutant tumors express stem cell markers.** (A-D) *lgl*^−/−^ T3 tumor mass (red) isolated from the host's abdomen and stained for various markers: NSC marker (Dpn, magenta; A), ganglion mother cell (GMC) marker (Pros, green; B), glial cell marker (Repo, gray; C) and neuronal cell marker (Elav, gray; D). (E-H) *pins^62/83^* T3 tumor mass (green) isolated from the host's abdomen and stained for various markers: NSC marker (Dpn, magenta; E), GMC marker (Pros, green; F), glial cell marker (Repo, gray; G) and neuronal cell marker (Elav, gray; H). For *lgl*^−/−^, the percentage of respective cells was determined by counting positively stained cells and normalizing to the DAPI-positive cells from several region of interests (ROIs). For *pins^62/83^*, the majority of the cells (in ROIs observed) were found to be negative for markers (Pros, Repo and Elav). Genotypes: (A-D) *w^11-18^*//*lgl^4/4^-mCD8-RFP* tumor (T3), (E-H) *w^11-18^*//*pins^62/83^-mCD8-GFP* tumor (T3).

Having established the serial transplantation tumor assay for two distinct NSC pathways, we could then perform comparative analyses at cellular and tissue levels. We began by investigating the cellular composition of *lgl^−/−^* and *pins^62/83^* mutant tumors using markers for stem, neuronal and glial cell differentiation. In *lgl^−/−^* T3 tumors, we found that >90% of tumor cells expressed the NSC marker Dpn, while only ∼5% expressed the ganglion mother cell (GMC) marker Prospero (Pros) (Fig. 3A,B). In addition to NSC and GMC markers, *lgl^−/−^* tumors also expressed differentiation markers associated with glial [Reversed polarity (Repo); <1% of cells] and neuronal [embryonic lethal abnormal vision (Elav); ∼5% of tumor cells] cell fates (Fig. 3C,D). This suggests that *lgl^−/−^* tumor cells comprise an overwhelmingly undifferentiated stem-cell population with only a small number expressing GMC markers, which display a differentiation bias toward neuronal lineages.

In contrast, *pins^62/83^* T3 tumors displayed a different cellular composition. Most (>99%) *pins^62/83^* tumor cells expressed the NSC marker Dpn, with no detectable nuclear staining for the GMC marker Pros (Fig. 3E,F). Similar to *lgl^−/−^*, *pins^62/83^* tumors lacked the glial marker Repo (Fig. 3G). However, unlike the *lgl^−/−^* tumor, *pins^62/83^* tumors also lacked the neuronal cell marker Elav (Fig. 3H). These findings suggest that although *pins^62/83^* and *lgl^−/−^* tumors originate from NSCs and share several tumorigenic properties, their cellular composition and features differ.

### NSC- and epithelial-derived tumors differ in secondary site dissemination

Given the success of our serial transplantations, which showed extensive tumor growth, we wondered whether these tumors could metastasize to various host tissues. Previous studies have shown that *lgl* mutant NSC-derived tumors transplanted into adult host flies indeed populated at distant sites, including the head capsule, thorax and legs (Gateff, 1978). Additionally, *lgl* mutant tumors in transplantation assays have been reported to invade host ovaries (Beaucher et al., 2007a,b). Although these findings indicate the metastatic potential of *lgl* mutant tumors, they provide limited information and cellular details regarding metastasized tumor cells in distant organs. Moreover, a comprehensive understanding of how distinct tumor types – originating from NSCs versus neoplastic epithelial cells – behave within the allograft transplantation model remains limited.

**Fig. 4.**
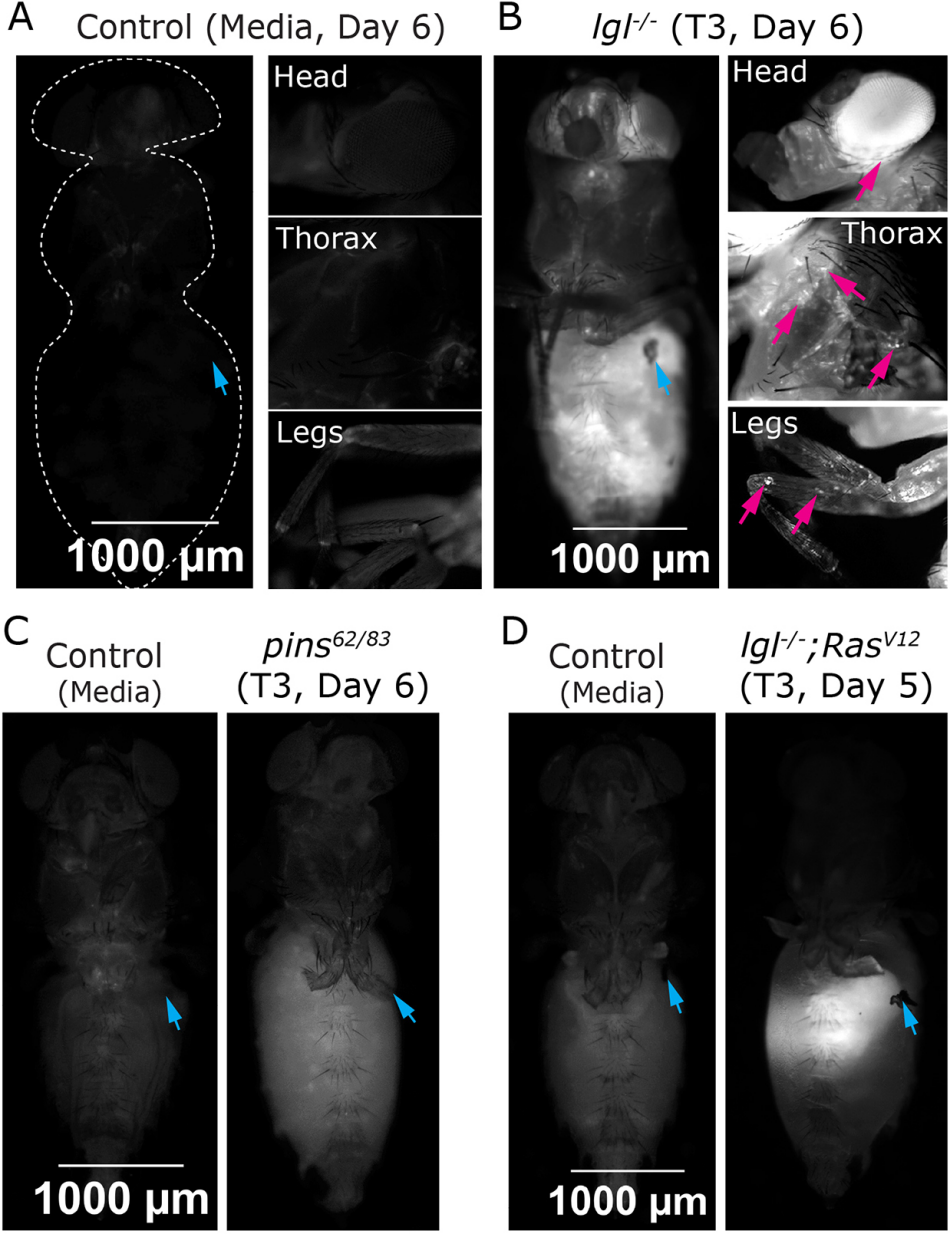
**Distinct metastatic behaviors of transplanted NSC- and epithelial-derived tumors.** (A) Representative fluorescence images of host flies 6 days after injection with control S2 medium, showing no detectable tumor growth in the head, thorax or appendages. (B) Host flies injected with *lgl*^−/−^ T3 tumors exhibit visible secondary tumor growths at Day 6, including prominent expansion in the head capsule, thorax and legs, indicating systemic dissemination and metastatic colonization of distant body sites. (C) Fluorescence images of host flies 6 days post-injection with control S2 medium (left) and *pins^62/83^* T3 tumors (right), showing minimal visible tumor burden. (D) Fluorescence images of host flies at Day 5 following injection with S2 medium (left) or *lgl*^−/−^ *Ras^V12^* T3 tumors (right), demonstrating extensive but localized tumor growth in the host's abdomen. Blue arrow, site of injection; magenta arrow, disseminated tumor cells. Genotypes: (B) *w^11-18^*// media and *w^11-18^*//*lgl^4/4^*-*mCD8-RFP* tumor (T3); (C) *w^11-18^*// media and *w^11-18^*//*pins^62/83^-mCD8-GFP* tumor (T3); and (D) *w^11-18^*// media and *w^11-18^*//*lgl^4/4^; Ras^V12^-mCD8-GFP* tumor (T3).

To probe these questions, we injected the T3 stage tumor mass (GFP or RFP labeled) into the abdomen of 3- to 4-day-old host flies and visualized tumor cells at various locations within the host fly 5-6 days after injection. Compared to the media-injected control, we found that, by Day 6, T3 *lgl* mutant tumor cells disseminated to multiple sites distant from the injection site (Fig. 4A,B). Further, we focused on various regions of the host flies and observed that tumor cells disseminated to multiple sites, such as the head capsule, thorax and appendages (Fig. 4B, magenta arrows). In contrast to *lgl^−/−^* tumors, *pins^62/83^* tumors showed a reduced capacity for migration to distant sites, with barely visible GFP signal at the distant site at Day 6 (Fig. 4C). This was potentially due to the loss of GFP in *pins^62/83^* tumors, making it challenging to visualize based on GFP signal alone (Fig. 2F; Fig. S2C and Fig. S3A).

To further investigate how the tumor origin influences metastatic potential, we examined an epithelial-derived tumor for its ability to disseminate to distant sites. We transplanted wing imaginal discs harboring the neoplastic genotype clones (*lgl^−/−^ Ras^V12^*) into adult host flies (Fig. S4A,B; Materials and Methods). Control discs (*Frt40*) did not give rise to tumors, while *lgl^−/−^ Ras^V12^* discs formed robust tumors in ∼75% of hosts within 5 days post-transplantation (Fig. S4C), as previously reported (Kim et al., 2021). Around 20% of these primary (T0) tumors exhibited spread to distant sites, including the head capsule, underscoring their invasive and metastatic behavior (Fig. S4C, magenta arrow).

Next, we evaluated the metastatic potential of these wing imaginal disc epithelial *lgl^−/−^ Ras^V12^* tumors in adult hosts. We applied the serial transplantation strategy previously established for NSC-derived tumors to obtain sufficient tumor samples by T3 (Fig. S4D; Materials and Methods). As expected, serial transplantation of epithelial *lgl^−/−^ Ras^V12^* tumors resulted in massive tumor growth, with tumors expanding rapidly with each successive round of transplantation, filling the host abdomen within just 5 days (Fig. S4E). Interestingly, unlike T0 tumors, which displayed a small degree of secondary site dissemination, these T1-T3 serially transplanted epithelial tumors remained strictly localized to the abdomen in 100% of cases in transplantation (Fig. S4E). Our analysis of *lgl^−/−^ Ras^V12^* T3 tumors revealed a stark contrast between NSC-derived and epithelial-derived tumors; epithelial-derived tumors appeared as distinctly localized tumor masses in the host's abdomen (Fig. 4D), whereas the NSC-derived tumors formed more diffuse clusters of tumor cells throughout the adult fly (Fig. 4B,C). Further, we probed the tumor progression and signs of tumor dissemination over days after injecting *lgl^−/−^ Ras^V12^* T3 tumors into adult host flies. Although rapid tumor expansion was evident in the abdomen by Days 2-4 and the tumor filled the entire abdomen by Day 8, no signs of tumor spread to secondary sites were observed (Fig. S4F).

Our findings demonstrate that tumor origin significantly influences metastatic potential. NSC-derived *lgl^−/−^* tumors showed robust dissemination to distant sites. In contrast, epithelial-derived *lgl^−/−^ Ras^V12^* tumors exhibited strong primary growth and remained localized in the abdomen (except for the T0 tumors). These results highlight a striking difference in metastatic behavior between NSC-derived and epithelial-derived tumors, emphasizing the role of tumor source in shaping metastatic capacity.

### NSC- and epithelial-derived tumors invade and colonize the host ovaries

Next, we investigated the metastatic potential of tumors originating from both NSCs and epithelial tissues by testing their ability to invade and colonize the host's internal organs. Prior studies have reported that NSC-derived tumors, particularly *lgl* and *brain tumor* (*brat*) mutants, can metastasize and invade host ovaries (Beaucher et al., 2007b). We expanded our study by performing a comprehensive analysis of the host's internal organs following the transplantation of a T3-stage tumor of NSC and epithelial origin.

**Fig. 5.**
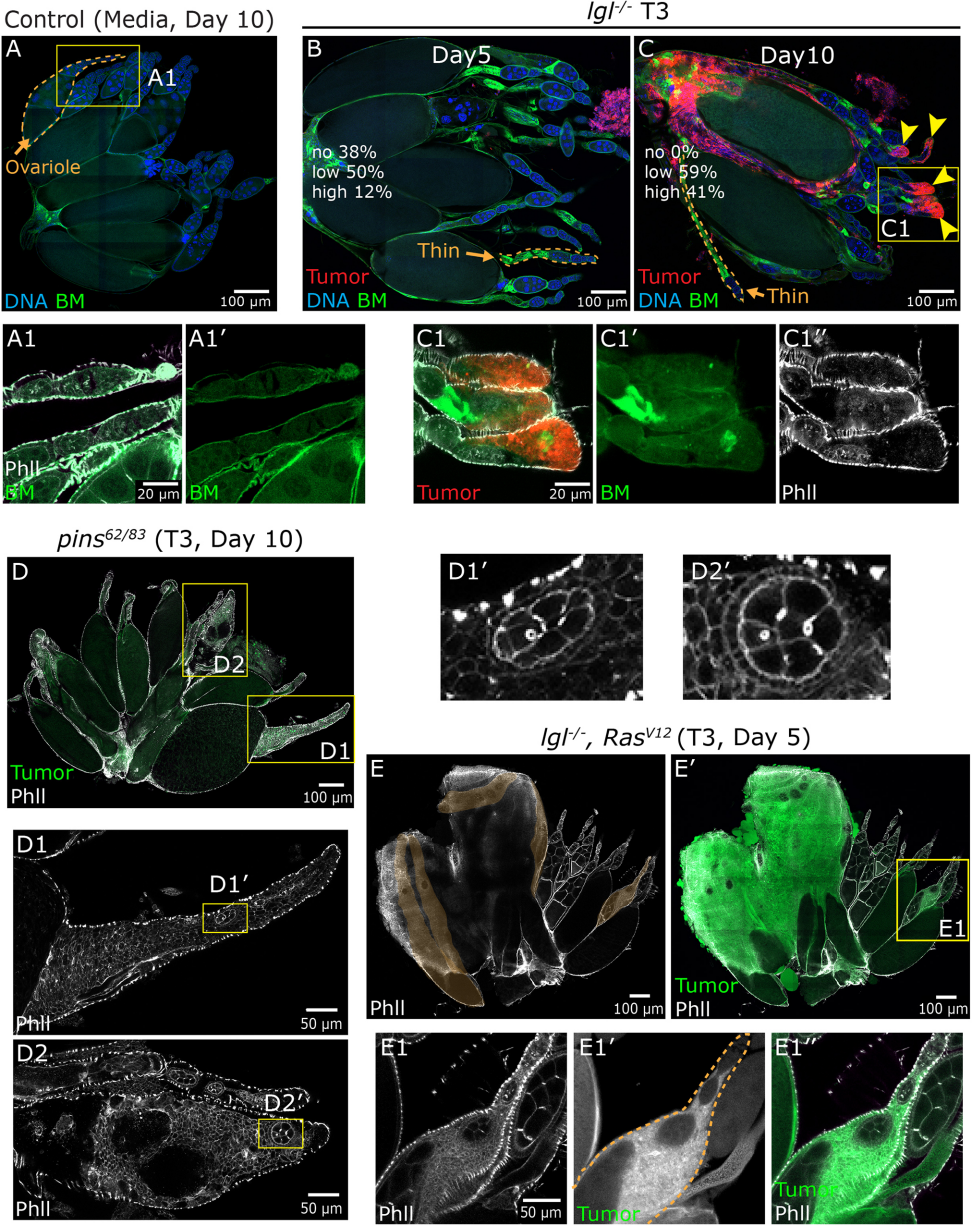
**NSC- and epithelial-derived tumors metastasize to the host ovaries.** (A,A1) Ovary isolated 10 days after S2 medium injection, marked for basement membrane (BM; Vkg-GFP, green) and stained with a nuclear/DNA marker (blue); a normal-sized ovariole is outlined in amber. (A1,A1′) Magnified image of a single ovariole stained with phalloidin (Phll) to show epithelial sheath (gray) and BM (green). (B) Ovary isolated 5 days (no invasion category) after *lgl*^−/−^ T3 tumor (red) injection, showing structural defects marked as thin ovarioles (amber dashed outline); text denotes the corresponding percentages of ovaries with varying degrees of tumor invasion at Day 5. (C,C1) Ovary isolated 10 days (high invasion category) after *lgl*^−/−^ T3 tumor (red) injection, showing multiple ovarioles invaded by tumor (yellow arrowheads) and structural defects marked as thin ovarioles (amber dashed outline); text denotes the corresponding percentages of ovaries with varying degree of tumor invasion at Day 10. (C1-C1″) Magnified image of ovarioles showing tumor (red) that has passed through the BM (green; C1′) and epithelial sheath (gray; C1″). (D-D2) Ovary (high invasion category) isolated 10 days after *pins^62/83^* T3 tumor (green) injection, showing tumor cells (GFP, green; phalloidin, gray) inside multiple ovarioles. (D1,D2) Magnified images of ovarioles from boxed areas in D stained with phalloidin (gray). (D1′,D2′) Magnified images from boxed areas in D1 and D2, showing egg chambers embedded within the tumor cluster inside the ovarioles. (E) Ovary isolated 5 days after *lgl*^−/−^ *Ras^V12^* T3 tumor (green), showing a massive tumor mass growing over a portion of the ovary marked by phalloidin (gray). Highlighted region (amber dashed outline) shows ovarioles embedded within the growing tumor. (E1-E1″) Magnified image of the boxed area in E′, with phalloidin staining marking the epithelial sheet (gray, E1), invaded tumor (amber dashed outline, E1′), and overlay image (E1″). Genotypes: (A) *Vkg-GFP*// media; (B,C) *Vkg-GFP*//*lgl^4/4^*-*mCD8-RFP* tumor (T3); (D) *w^11-18^*//*pins^62/83^-mCD8-GFP* tumor (T3); and (E) *w^11-18^*//*lgl^4/4^; Ras^V12^*-*mCD8-GFP* tumor (T3).

To begin, we isolated host ovaries at Day 5 and Day 10 after transplanting the *lgl^−/−^* T3 tumor. Our results indicated that tumors spread to the host ovaries as seen by the presence of tumor cells around and inside ovarian tissue. We also noticed that ovaries contained significantly deformed and thinner ovarioles (evidenced by the loss and collapse of egg chambers, outlined in amber in Fig. 5A-C) on Day 5 and Day 10 after tumor injection compared to media-injected control ovaries. In addition to the tumor spreading to the ovarian tissues, several instances showed that tumor cells invaded individual ovarioles. To thoroughly investigate the extent of tumor invasion, we divided ovaries into three broad categories. (1) No invasion: the tumor surrounded the ovaries but presented no sign of tumor invasion within the ovarian tissue (Fig. 5B; Day 5). (2) Low invasion: the tumor surrounded the outer ovaries but only invaded one or two ovarioles. (3) High invasion: a large amount of tumor enwrapped the ovaries and invaded many ovarioles (Fig. 5C, yellow arrowheads; Day 10). On Day 5, 38% of the ovaries were ‘no invasion’, 50% were ‘low invasion’, and 12% were ‘high invasion’. The extent of ovarian invasion by tumor increased at Day 10, with 100% of ovaries showing signs of invasion: 59% low invasion and 41% high invasion. This suggests that the no invasion category on Day 5 eventually experienced tumor high tumor invasion within a few days.

Next, we investigated whether the tumor could pass through the tissue layers surrounding the *Drosophila* ovaries. To visualize these structures, we used F-actin to label the peritoneal sheath (smooth muscle cells) surrounding the ovaries and the epithelial layers enveloping individual ovarioles (Fig. 5A1). Additionally, we marked the basement membrane (BM) using Collagen-IV-GFP [Viking (Vkg)-GFP], which encases individual ovarioles (Fig. 5A1′). Our analysis revealed that *lgl^−/−^* T3 tumor cells actively invaded through the peritoneal sheath, breached the epithelial layer and crossed the BM (Fig. 5C, yellow arrowheads; Fig. 5C1-C1″), as had been seen before (Woodhouse et al., 1998). Notably, tumor cells formed colonies within the ovarioles, resulting in displaced and dying egg chambers, causing significant deformation of the ovarioles (Fig. 5C, yellow arrowheads; Fig. 5C1). However, we do not have evidence that tumor cells breach the BM of the individual follicle or egg chamber.

These results confirmed that tumor cells had disseminated and invaded host ovaries. Next, we tested whether tumors were also successful in seeding colonies within the ovarian tissue by staining for mitotic cells using antibody against phospho-histone 3 (PH3) (Fig. S5A-A1′). Our results showed that tumor cells had not only invaded the ovarian tissue but also proliferated, as indicated by PH3-positive tumor cells in both low invasion (Fig. S5B-B1′) and high invasion (Fig. S5C-C2″) categories. Particularly, we noticed several PH3-positive tumor cells surrounding the egg chamber in the high invasion category, indicating that tumor cells had not invaded these ovarioles and actively proliferated. Overall, these results from ovaries suggest that *lgl* mutant tumors exhibit essential signatures of forming tumor metastatic colonies at the secondary organ site.

We then analyzed the metastatic potential of *pins^62/83^* T3 tumors by examining their ability to invade host ovaries. By Day 10 post-transplantation, we observed a marked reduction in ovariole size, with most samples containing much thinner ovarioles than controls (Fig. 5D; Fig. S6A,B). Notably, GFP expression was largely lost in *pins^62/83^* tumor cells associated with the ovary, requiring phalloidin staining for visualization of tumors based on their morphology (Fig. 5D; Fig. S5B). Similarly to *lgl^−/−^* tumors, *pins^62/83^* tumors exhibited variable degrees of ovarian metastasis quantified for Day 10: (1) no invasion, 47%; (2) low invasion, 42% (Fig. S5B); and (3) high invasion, 11% (Fig. 5D). In high invasion cases, tumor cells were detected within multiple ovarioles, forming bulging structures indicative of secondary tumor colonies (Fig. 5D-D2). Unlike *lgl^−/−^* tumors, which displaced egg chambers, *pins^62/83^* tumors embedded within them, forming structurally distinct metastatic colonies (Fig. 5D1-D2′). Although both tumor types arise from NSCs, these findings highlight distinct metastatic behaviors, suggesting that genetic differences in the tumor origin significantly influence metastatic patterns and organ colonization.

We next examined secondary metastasis to the ovaries following transplantation of *lgl^−/−^ Ras^V12^* epithelial tumors. Unlike the more diffuse tumor pattern seen with NSC-derived tumors, *lgl^−/−^ Ras^V12^* T3 tumors formed compact, discrete masses in the host abdomen (Fig. 4D). We dissected the GI tract, ovaries and tumor mass complexes from host flies 5 days post-transplantation to assess tumor interactions with host tissues. Gross analysis revealed that tumors expanded to envelop and enwrap the GI tract and ovaries (Fig. S6C,C′).

Consistent with earlier reports on epithelial tumor model (*aPKC^−/−^ Ras^V12^*) (Kim et al., 2021), we observed that ∼42% of ovaries exhibited significant thinning of ovarioles within 5 days of tumor transplantation compared to the control (this was independent of tumor metastasis to the ovary) (Fig. S6D-F). However, a larger subset (58%) of ovaries did not display signs of ovariole thinning, even when surrounded by an enormous tumor mass (Fig. 5E). Closer examination revealed that the tumor fused with and embedded several ovarioles, evident with phalloidin staining (Fig. 5E, amber dashed outline; Fig. S6E). We noticed that, in some instances, tumor cells breached the ovarian surface, infiltrating the cellular layers and forming secondary tumor colonies within the ovarioles (Fig. 5E1-E1″, yellow boxed area in Fig. 5E′). This indicated that neoplastic epithelial (*lgl^−/−^ Ras^V12^*) tumors not only peripherally enwrapped the ovarian tissue but also passed through the peritoneal sheath and the epithelial cell layer that surrounds individual ovaries and ovarioles, which was not reported in previous studies. These findings were similar to those observed with NSC-derived tumors, indicating that both tumor types independent of their properties display capacity to invade and potentially seed secondary metastases in host ovaries.

### Metastasis to the GI tract

**Fig. 6.**
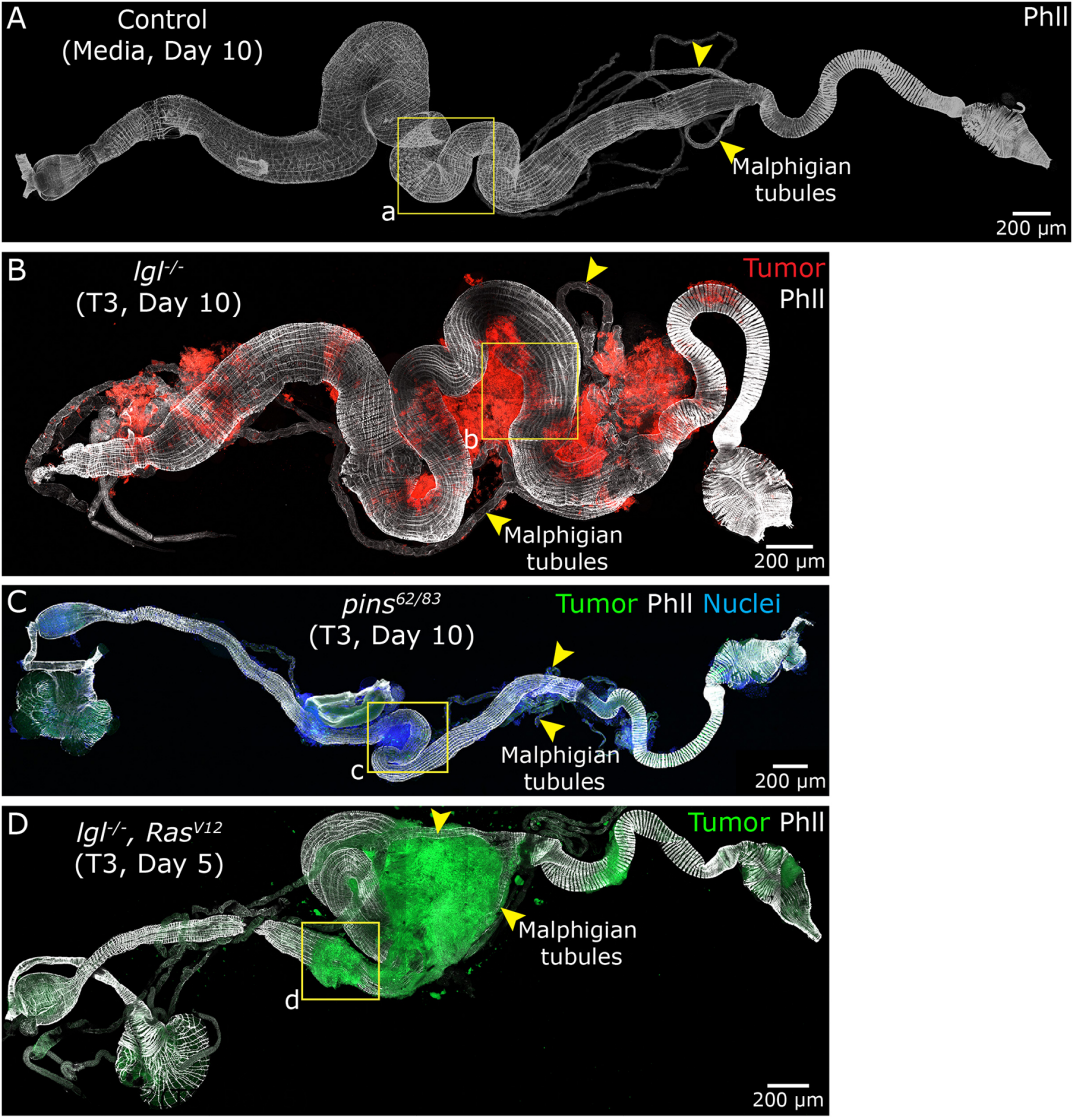
**NSC- and epithelial-derived tumors metastasize to the gastrointestinal tract but fail to pass through the cellular layers.** (A) Gastrointestinal (GI) tract dissected from control host flies 10 days after S2 medium injection; phalloidin staining (gray) marks visceral muscles (boxed area a is shown magnified in Fig. S7A). (B) GI tract isolated 10 days after *lgl^−/−^* T3 tumor injection (red); tumor cells are observed decorating the midgut and hindgut regions and GI visceral muscles (gray) (boxed area b is shown magnified in Fig. S7B). (C) GI tract dissected 10 days post-injection with the *pins^62/83^* T3 tumors (green); tumor cell nuclei (blue) and visceral muscles (gray) are also shown (boxed area c is shown magnified in Fig. S8B-D). (D) GI tract isolated 5 days after injection with *lgl^−/−^ Ras^V12^* T3 tumors (green); visceral muscles (gray) are also shown (boxed area d is shown magnified in Fig. S8F). Yellow arrowhead, Malpighian tubules. Genotypes: (A) *Vkg-GFP*//media; (B) *Vkg-GFP*//*lgl^4/4^*-*mCD8-RFP* tumor (T3); (C) *w^11-18^*//*pins^62/83^*-*mCD8-GFP* tumor (T3); and (D) *w^11-18^*//*lgl^4/4^; Ras^V12^*-*mCD8-GFP* tumor (T3).

We next examined the host GI tract for evidence of tumor metastasis. To visualize the GI tract, we used phalloidin staining and isolated the GI tracts from control flies injected with media (Fig. 6A) and host flies injected with *lgl^−/−^* T3 tumors, 10 days post-injection (Fig. 6B). We found that *lgl^−/−^* tumors decorated the midgut and hindgut regions of the GI tract. Closer inspection of the tumor-associated region (Fig. 6, yellow boxed areas a and b) revealed that tumor cells did not breach the GI wall, which maintained an apparent intact GI muscle (phalloidin staining) and BM (Vkg-GFP) layers (Fig. S7A,B), with no evidence of tumor cells in the gut lumen (Fig. S7B1). However, we did observe that the tumor-associated GI regions were distorted, with noticeable lumen constriction at the tumor-contact sites (Fig. S7B1-B2′). These findings suggest that although the tumor encapsulated the gut externally and caused significant distortion, it failed to pass through the GI tissue layers.

Similarly to *lgl^−/−^* tumors, *pins^62/83^* T3 tumors decorated the midgut and hindgut regions of the GI tract, although a lesser degree of tumor burden decorated the GI tract compared to *lgl^−/−^* tumors (Fig. 6C). As observed in the ovaries, most *pins^62/83^* tumor cells lost GFP expression and were detected using nuclear staining instead (Fig. 6C). Closer examination of tumor-associated regions (Fig. 6C, yellow boxed area c) revealed that although the tumor distorted the gut lumen (Fig. S8A,B), no tumor cells were observed breaching the GI wall and were absent in the gut lumen (Fig. S8C,D). These results suggest a shared pattern of metastasis between *lgl* and *pins* mutant tumors in targeting the GI tract.

Next, we tested *lgl^−/−^ Ras^V12^* T3 tumors for signs of metastasis in the host GI tract. We found that a massive tumor surrounded the GI tract and grew while attached to the midgut and hindgut, and surrounded the Malpighian tubules (Fig. 6D). Upon closer examination of the tumor-associated area (Fig. 6C, yellow boxed area d), it was evident that the tumor attached to the periphery of the GI tract and distorted the lumen, but failed to pass through the GI wall and into the lumen (Fig. S8E-F1′). This was similar to what we observed with NSC-derived tumors.

The results with these three tumor types, of two different origins (NSCs and epithelial tissue), showed a similar response in metastasizing to the adult fly GI tract and starkly contrasted with results for the ovary (Fig. 5). This suggests an inherent property of the fly GI tract in responding to metastatic tumors, whereby tumors spread, closely associated with and deformed the GI walls but failed to breach the cellular layer and BM.

### Metastasis to the fly brain

**Fig. 7.**
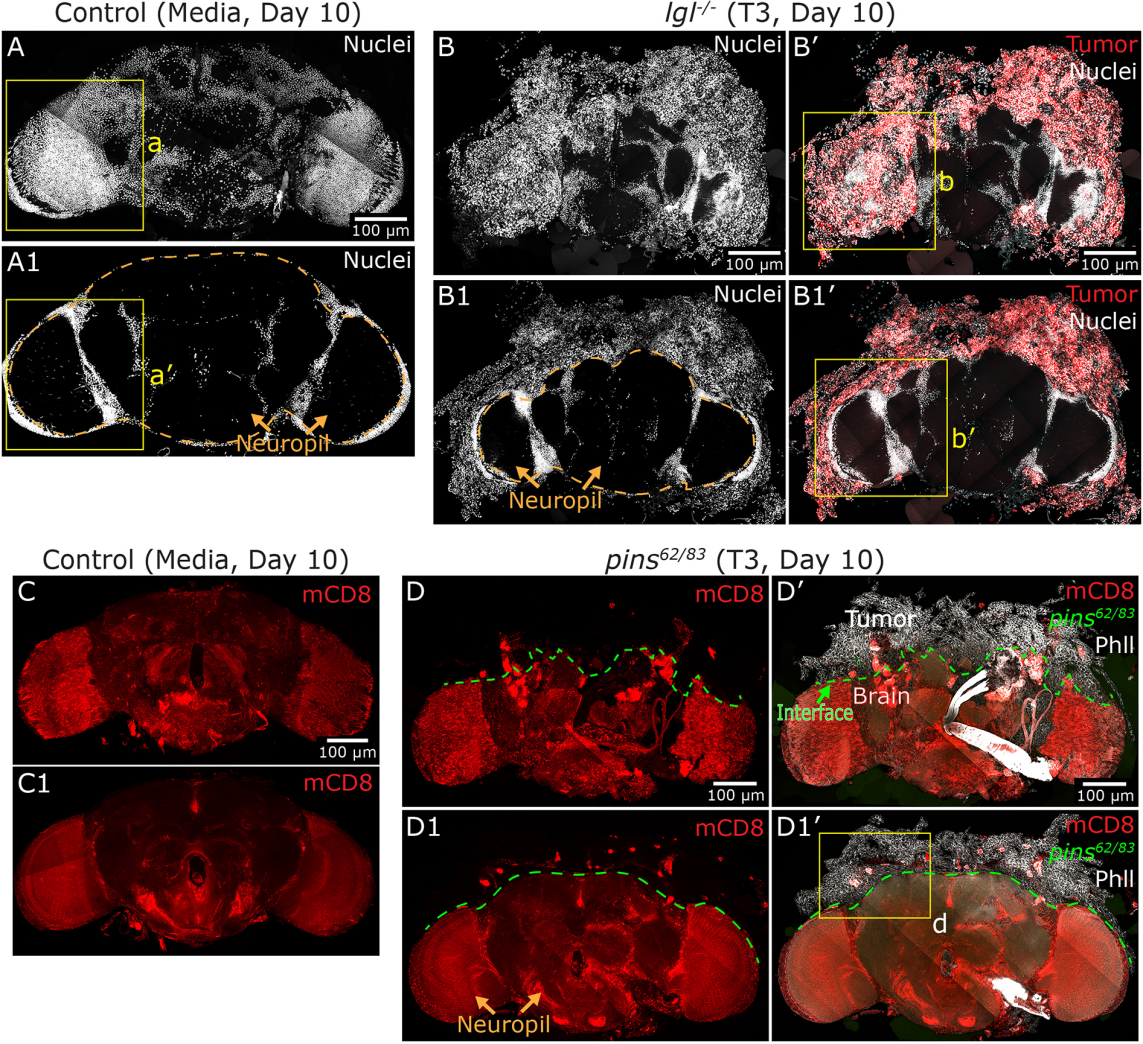
***lgl* and *pins* mutant tumors metastasize to adult fly brain.** (A,A1) Adult fly brain isolated from control host flies 10 days after S2 medium injection; nuclear staining marks brain cells (gray). Surface *z*-section (A) and middle *z*-section (A1) showing neuropil region (amber dashed outline and arrows). Boxed areas a and a′ are shown magnified in Fig. S9A,A1. (B-B1′) Brain isolated 10 days after *lgl^−/−^* T3 tumor injection. (B,B′) Surface *z*-section showing tumor cells (red) decorating the brain surface (marked by nuclei, gray). (B1,B1′) Middle *z*-section showing cortical deformation by tumor and absence of tumor from the neuropil region (amber dashed outline and arrows). Boxed areas b and b′ are shown magnified in Fig. S9B,B1. (C,C1) Control brain expressing mCD8-RFP (red) isolated 10 days after S2 medium injection. (D-D1′) Brain (red) isolated 10 days after *pins^62/83^* T3 tumor injection (GFP and marked by phalloidin, gray). (D,D′) Surface *z*-section showing tumor cells (phalloidin, gray) decorating the brain surface (red; green dashed outline marks the deformation of the brain). (D1,D1′) Middle *z*-section showing cortical deformation by tumor and absence of tumor from the neuropil region (amber dashed outline and arrows). Boxed area d is shown magnified in Fig. S9E. Genotypes: (A,B) *w^11-18^*// media and *w^11-18^*//*lgl^4/4^*-*mCD8-RFP* tumor; (C,D) *tub-Gal4::mCD8-RFP*// media and *tub-Gal4::mCD8-RFP*//*pins^62/83^*-*mCD8-GFP* tumor (T3).

In addition to the tissues described so far, we observed that *lgl^−/−^* NSC-derived tumors exhibited a strong affinity for the host fly's head, with a pronounced spread to the head capsule as early as day 2 (Fig. 2D; Fig. S2A,B). This led us to wonder whether the tumor truly invaded the adult fly brain. To assess this, we isolated brains from host flies on Day 10 after injecting the *lgl^−/−^* T3 tumor, and tumors were abundantly seen in the head capsule. Our results revealed that the tumor metastasized to the brain, grew to a considerable size and effectively enwrapped the brain surface (Fig. 7B). As these metastasized tumors grew, they deformed the cell cortex region of the central brain and the optic lobes (Fig. 7B-B1′), compared to the organized and aligned cellular cortex region of control brains (Fig. 7A,A1). However, we did not notice any sign of tumor passing across the brain cell cortex, as no tumor cells were found in the neuropil region (Fig. B1, amber dashed outline; Fig. S9A-B1′). Further, a closer analysis of the sites that appeared to be invasion zones where the tumor and brain interfaced (Fig. 7D, green dashed outline; Fig. S9C) showed deformed brain cell cortex (Fig. S9C″, yellow arrowheads). These results suggested that *lgl^−/−^* tumors displayed a strong affinity for the fly brain; however, these tumors grew profusely and deformed the outer surface but failed to pass through the cellular layer. This is similar to leptomeningeal metastasis, where metastasized tumors grow in close association with the meningeal layer without any significant infiltration to the brain parenchyma (Freret and Boire, 2024; Remsik and Boire, 2024).

To test whether tumor size passively increased in size by migration of additional tumor cells from the primary site, or whether the tumors in fact grew at the secondary site by undergoing proliferation as expected for metastatic tumors, we stained for mitotic cells using anti-PH3 antibody. Interestingly, our results revealed that tumor cells (labeled by GFP expressed under *png-Gal4*) that spread to the brain surface were indeed proliferative, as evident by several PH3-positive cells (Fig. S9D-D1′), including cells in direct contact with the brain surface (Fig. S9D1,D1′). These results indicate that although tumors did not penetrate deep into the neuropil region, they did actively grow and deformed the brain surface, similar to meningeal metastasis (Abeysundara et al., 2025).

Unlike *lgl^−/−^* tumors, which prominently filled the head capsule, *pins^62/83^* tumors displayed minimal GFP signal in this region, likely due to GFP loss in tumor cells (Fig. 2F; Fig. S2C). To assess metastatic spread to the brain, we dissected host brains 10 days post-injection with *pins^62/83^* T3 tumors. Despite lacking a GFP signal, a significant tumor burden was observed in the brain via phalloidin staining (Fig. 7D). Co-labeling the host brain with mCD8-RFP revealed that, similar to *lgl^−/−^* tumors, *pins^62/83^* tumors closely associated with and distorted the brain surface (Fig. 7D,D1, green dashed outline), unlike the uniform brain surface of controls (Fig. 7C,C1). *pins^62/83^* tumors also grew peripherally attached onto the brain surface, with no evidence of tumor cells in the neuropil region of the host brain (Fig. 7C1). Furthermore, analysis of the tumor–brain interface at the invasion zones (Fig. 7D1′, boxed area d) revealed that the *pins* mutant tumors were tightly associated with the brain surface, as shown with phalloidin staining (Fig. 7D1′, boxed area d; Fig. S9E). These results were similar to what we observed with *lgl^−/−^* tumors; however, despite differences in cell type composition, *lgl* mutant tumors exhibited neuronal characteristics, with most tumor cells remaining in a stem cell-like state (Fig. 3). In contrast, the *pins* mutant tumors consisted predominantly of stem cells lacking neuronal or glial markers (Fig. 3).

**Fig. 8.**
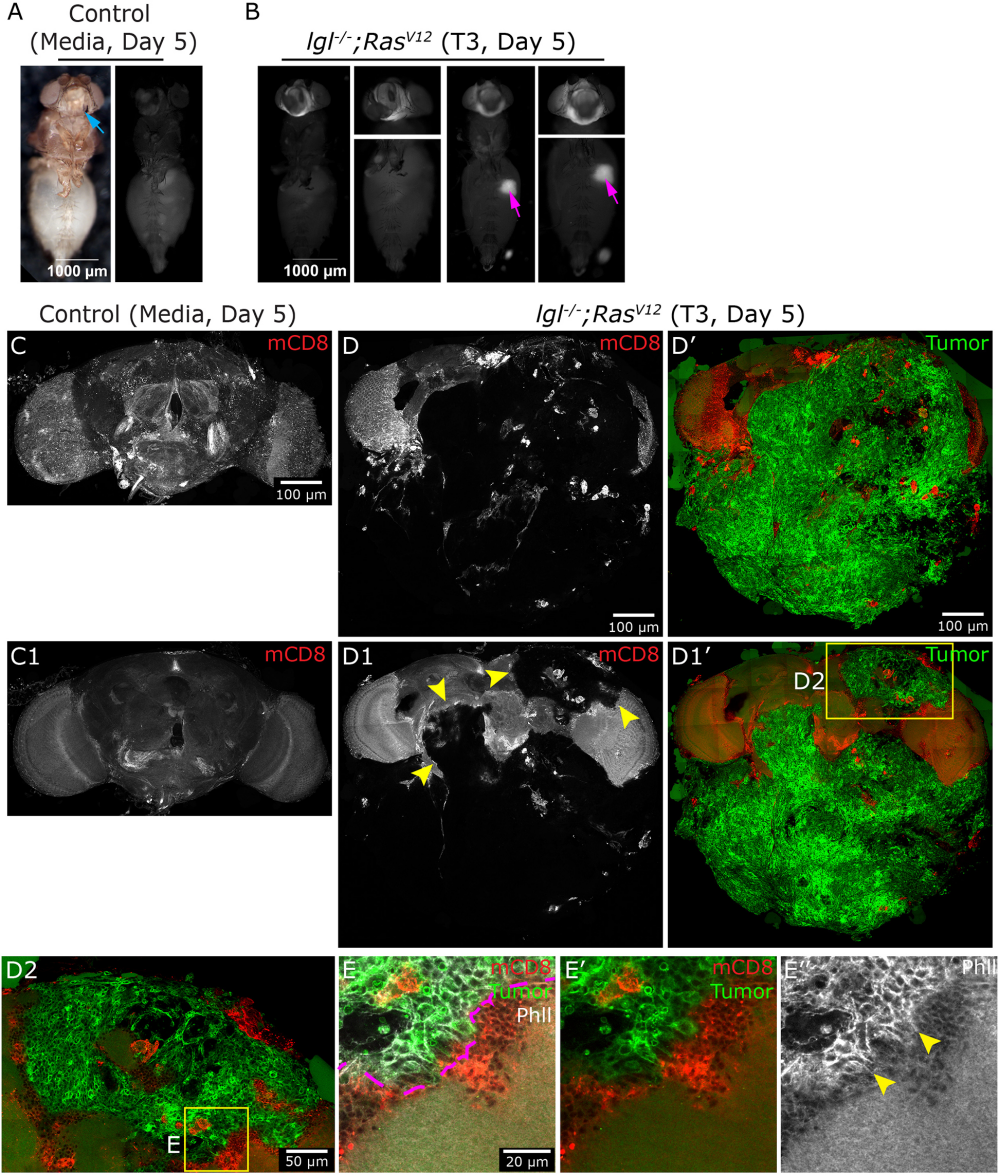
**Epithelial-derived tumors metastasize to the adult brain.** (A) Bright-field and fluorescence image of the adult fly 5 days after injection with S2 medium (control) in the mouth part ‘proboscis’ (blue arrow). (B) Fluorescence image of the adult fly at Day 5 after injection with *lgl^−/−^ Ras^V12^* T3 tumor in the mouth part (tumor passed to the abdomen, magenta arrows). (C,1) Control brain expressing mCD8-RFP (red) isolated 10 days after S2 medium injection. (D-D1′) Brain (red) isolated 5 days after *lgl^−/−^ Ras^V12^* T3 tumor proboscis injection (GFP and marked by phalloidin, gray). (D,D′) Surface *z*-section showing tumor (green) deforming the brain surface (red). (D1,D1′) Middle *z*-section showing significant brain deformation by tumor (yellow arrowheads). (D2) Region encompassing the brain (red)–tumor (green) interface. (E-E″) A further magnified image showing the brain (red)–tumor (green) interface (magenta dashed outline), with phalloidin staining marking the tumor cellular projections (gray, yellow arrowheads). Genotypes: (A) *w^11-18^*// media; (B) *w^11-18^*//*lgl^4/4^; Ras^V12^*-*mCD8-RFP* tumor; (C) *tub-Gal4::mCD8-RFP*// media; and (D) *tub-Gal4::mCD8-RFP*//*lgl^4/4^; Ras^V12^*-*mCD8-GFP*.

It is possible that NSC-derived (both *lgl* and *pins*) tumors display affinity for the host's brain owing to their shared cellular origin. To address this, we used the *lgl^−/−^ Ras^V12^* epithelial tumor model as a comparison; however, as previously noted, *lgl^−/−^ Ras^V12^* tumors remained confined to the host abdomen, with no detectable signal in the head capsule (Fig. 4D). To overcome this obstacle, we optimized a method for injecting the tumor mass into the proboscis, avoiding damage to the head or mouthparts (Fig. 8A, blue arrow; Materials and Methods). This approach overcame the lack of tumor dissemination to the fly head and enabled targeted tumor transplantation in the head. With this approach, the *lgl^−/−^ Ras^V12^* T3 tumor grew considerably by Day 5; however, the tumor remained confined mainly to the head, with rare instances of spreading beyond the head region (Fig. 8B, magenta arrows). Upon isolating the host brain on Day 5 post-T3 tumor injection, we observed a large, discrete tumor mass in the head capsule (Fig. 8D). Despite remaining localized, the tumor had a significant impact on brain morphology, causing severe distortion and fragmentation of the brain surface, and, even in deeper middle sections, effectively replacing the brain tissue (Fig. 8C-D1′, yellow arrowheads). We focused on invasion-like zones to test tumor involvement with the host brain (Fig. 8D2). This revealed, that instead of superficial engagement with the brain, tumor closely associated with brain tissue, as evident at the tumor–brain interface (Fig. 8E, marked by magenta dashed outline), showing the tumor's cellular projection by membrane marker and phalloidin staining in tight contact with brain tissue (Fig. 8E-E″).

Collectively, findings from all three tumor models reveal distinct metastatic patterns to the *Drosophila* brain. Tumors derived from NSCs display peripheral sheet-like growth enwrapping the host's brain and are closely associated with the brain's cellular cortex. In contrast, epithelial-derived tumors exhibit expansive growth and appear to penetrate deeper brain regions. However, additional future work that directly injects NSC tumors into the head would be required for direct comparison with the epithelial-derived tumors. Notably, the *Drosophila* brain is encased by a surface-level blood–brain barrier (BBB), unlike the mammalian BBB, which comprises capillaries infiltrating the brain parenchyma (Contreras et al., 2024; Limmer et al., 2014). These observations provide a valuable framework for investigating tumor–BBB interactions during brain metastasis.

## DISCUSSION

This study establishes allograft transplantation as a powerful and accessible platform to investigate metastatic tumor progression and tumor-host interactions in adult *Drosophila*. To begin, we employed *lgl* mutant brains to generate T0 tumors, which were then serially transplanted to propagate tumors across multiple generations. By leveraging serial transplantation of *lgl* mutant brain-derived tumors, we address major limitations of primary tissue transplantation – namely, low tumor induction frequency, technical barriers and extended tumor latency. This strategy not only enables reliable tumor propagation across multiple transplantation generations but also facilitates detailed characterization of temporal dynamics during metastatic progression. Notably, we identify the T3-T4 transplantation stages as optimal for experimental scalability, during which tumors robustly expand and disseminate to distant host tissues over a defined temporal window spanning 1-14 days post-transplantation. Together, this approach enables previously unattainable large-scale investigations of metastatic behavior in adult *Drosophila* by (1) establishing a robust and scalable allograft transplantation model; (2) standardizing serial transplantation protocols and stages to reliably propagate tumors across multiple generations, thereby increasing reproducibility and experimental throughput; and (3) capturing the temporal dynamics of tumor progression and dissemination *in vivo*, from initial engraftment to metastatic spread across secondary organ sites.

To assess the versatility of this model, we extended our approach to tumors derived from a second NSC mutant, *pins*. Despite distinct phenotypic differences between *pins* and *lgl* mutants at the NSC level, *pins*-derived tumors showed comparable tumor induction and propagation efficiency through serial transplantation. These tumors also disseminated to secondary sites, although the extent of spread appeared reduced, likely due to the gradual loss of the fluorescent reporter, which limited our ability to track tumor cells over time. Nonetheless, *pins* and *lgl* tumors showed similar properties in terms of spreading to distant sites, highlighting the broader applicability of this system to diverse tumor genotypes.

Further, we extended our analysis to epithelial tumors to evaluate whether this approach could be generalized beyond NSC-derived tumors. Like NSC tumors, epithelial tumors were successfully propagated through serial transplantation. However, they exhibited more restricted dissemination, with only limited metastasis to distant sites. Interestingly, despite these differences in dissemination capacity, both NSC- and epithelial-derived tumors demonstrated the ability to invade secondary organ sites, suggesting that certain features of metastatic invasion are conserved across tissue types.

Collectively, our study bridges the gap between traditional *Drosophila* tumor models, which have focused on primary tumor growth in larval tissues (Brumby and Richardson, 2003; Pagliarini and Xu, 2003), and the need for adult models that allow temporal and spatial dissection of metastatic behavior. These adult *Drosophila* allograft models enable real-time visualization of tumor–host interactions at cellular resolution, including tumor invasion and organ colonization. These features offer valuable parallels to mammalian metastasis, particularly with respect to ovarian and brain tumor invasion, establishing *Drosophila* as a workable platform for uncovering basic cellular mechanisms driving tumor–host interaction at secondary metastasis sites.

### Overcoming allografting hurdles by serial transplantation

Allograft transplantation has long been a foundational technique in tumor biology (Beadle and Ephrussi, 1935, 1936a,b; Gateff and Schneiderman, 1969). However, its broader application, particularly in studying tumor metastasis, has been limited by significant technical challenges, including the difficulty of transplanting primary tissues, the low efficiency of tumor induction and extended latency before tumor development. These limitations have traditionally constrained the scalability and reproducibility of such approaches. To overcome these obstacles, previous studies have employed serial transplantation, whereby tumor tissue is propagated across successive host generation (Beaucher et al., 2007a). This strategy not only generates sufficient tumor material for downstream analyses but has also been shown to enhance the metastatic potential of both NSC-derived tumors and those of *Drosophila* larval salivary gland imaginal rings (Cong et al., 2025). In our work, we build on these earlier studies by streamlining and formalizing the serial transplantation procedure into clearly defined stages. We identify T2 tumors as a reliable starting point for expansion, with T3-stage tumors yielding sufficient mass for transplanting ∼40-50 adult host flies per donor, marking a critical threshold for scalability in large-scale studies. Whereas we used WT hosts for serially propagating the tumor over generations, previous studies incorporated *ovo^D^* flies, which lack ovaries, as donor hosts to further increase the amount of extractable tumor tissue per fly (Beaucher et al., 2007a). In the future, utilizing *ovo^D^* flies in combination with our serial transplantation strategy would be useful to scale up tumor mass sufficiently for injection into an even larger number of host flies.

One frequently raised concern regarding allografting involves the potential for host injury during transplantation, which may provoke immune responses that confound tumor progression analyses (Chen et al., 2023). However, our observations suggest that serially transplanted tumor tissues, being smaller and more manageable, can be introduced with less invasive manipulation; however, whether this results in lesser injury and immune response needs to be tested. Nevertheless, we found serial transplantation to be efficient for transplanting into a large number of host flies in a short period of time without any significant host lethality associated with the transplantation procedure.

### Origin-dependent differences in tumor progression in the allograft model

In addition to intrinsic genetic alterations, tumor progression from initiation to metastasis is strongly shaped by the tissue microenvironment, secreted signals, immune responses and overall host physiology. Notably, several NSC genotypes that do not form tumors *in situ* undergo robust tumor growth when larval brains are transplanted into the adult *Drosophila* abdomen (Caussinus and Gonzalez, 2005). This suggests that allografting aids tumor induction either by extending the proliferative window or by exposing the transplanted tissue to a host-derived milieu that promotes tumor initiation. A striking example is the *pins* mutant, which exhibits limited proliferation *in situ* but undergoes symmetric divisions and forms aggressive tumors upon transplantation (Caussinus and Gonzalez, 2005). In support of the host contribution, previous studies have shown that this transformation is driven by host-mediated activation of the mTOR and PI3K signaling pathways, essential for supporting the symmetric division of the *pins* mutant NSCs (Rossi and Gonzalez, 2012). Our findings reveal another aspect of the *pins* mutant tumor – likely enhanced genomic instability, potentially adding another layer of tumorigenic drive (Fig. S3).

The nature of genetic lesions plays a critical role in shaping tumorigenic pathways. Our study compared two distinct NSC-derived tumor models – *lgl* mutants, which disrupt cell polarity, and *pins* mutants, which impair the mitotic spindle checkpoint. We found that *lgl* mutant tumors, unlike *pins* mutant tumors, do not appear to exhibit genomic instability, suggesting divergent mechanisms of tumorigenesis. *lgl* NSCs are intrinsically pro-tumorigenic owing to loss of cell polarity, leading to a higher rate of symmetric divisions and the capacity to drive brain overgrowth *in situ* (Gateff and Schneiderman, 1974; Lee et al., 2006). This tumorigenic potential is further amplified upon allograft transplantation, likely through interactions with yet unknown host factors. In contrast, *pins* mutant tumors possibly involve a combination of defective asymmetric cell division and mitotic errors, resulting in the accumulation of abnormal stem cell proliferation and genomic instability that, in combination with host factors, drives tumorigenesis. These findings highlight how different genetic drivers can cooperate with the host environment to influence distinct modes of tumor progression.

In support of distinct tumor trajectories, our findings also reveal slight differences in the cellular composition of *lgl* and *pins* mutant tumors. Although *pins* tumors primarily express markers of NSCs, a significant fraction of *lgl* tumor cells express GMCs and differentiated neuronal cell markers, along with a minor population of glial cells, contrary to previous reports (Beaucher et al., 2007a) (Fig. 3). These differences underscore the possibility that specific genetic lesions influence tumor initiation and growth and affect lineage specification within the tumor. However, how these variations in cellular composition contribute to tumor behavior and progression remains unclear. Future studies comparing tumors arising from distinct genetic disruptions in NSCs – such as polarity, mitosis, or differentiation – will provide deeper insight into the cellular and molecular mechanisms driving tumor heterogeneity, findings relevant to understanding cancer stem cells' proliferation and progression. Notably, the allograft transplantation system offers a unique opportunity to dissect these pathways, as it allows for independent manipulation of host genotype and tumor origin, thereby enabling functional analysis of tumor–host interactions *in vivo*.

The majority of allograft transplantation studies in *Drosophila* have focused on NSC-derived tumors. Much less is known about the behavior of epithelial or tumors originating from different tissue types. Broadly, how the cell of origin influences metastatic potential remains underexplored. Our findings reveal a striking contrast between NSC- and epithelial-derived tumors. Although *lgl^−/−^ Ras^V12^* neoplastic epithelial tumors are known to exhibit aggressive invasive behavior during larval stages (Brumby and Richardson, 2003; Pagliarini and Xu, 2003), in the adult host, they remained largely localized and failed to disseminate to distant sites. In contrast to the *lgl* mutant tumors, NSC-derived tumors exhibited rapid and robust metastatic spread, reaching distant organs within 2-4 days post-transplantation and forming prominent tumor masses (Fig. 4). These observations underscore the importance of tumor cell origin in shaping metastatic behavior and suggest that intrinsic cellular programs differentially respond to the host environment during tumor progression.

### Allograft method for modeling metastasis to secondary sites in *Drosophila*

*Drosophila* has long served as a powerful model for studying mechanisms of local tumor invasion, particularly using larval neoplastic tumors of epithelial origin (Brumby and Richardson, 2003; Pagliarini and Xu, 2003). Owing to its well-characterized tissue architecture and sophisticated genetic tools for cell type-specific manipulation, *Drosophila* is also well suited for investigating tumor interactions at distant, secondary sites. The evolutionary conservation of key cellular processes involved in migration and invasion provides a valuable opportunity to uncover fundamental mechanisms underlying metastasis and tumor–host interactions. However, the field has been limited by a lack of models that robustly form organ metastases. Building on previous work, we have addressed this gap by optimizing a serial transplantation protocol that enables reproducible tumor engraftment, spread and colonization of secondary organs. Our defined transplantation approach allows for temporal control and time-series analyses of metastatic progression in adult flies, enabling high-resolution imaging of metastatic events at the secondary host sites.

Consistent with previous studies, we found that *lgl* mutant tumors are capable of invading the host ovary. By leveraging T3-stage tumors, we examined the temporal progression of this metastatic behavior and observed that invasion peaked around Day 10 post-transplantation. As reported earlier, *lgl* tumor cells infiltrate multiple ovarian tissue layers (Beaucher et al., 2007a), traversing the peritoneal sheath, epithelial sheath and the BM surrounding the ovarioles (Fig. 5). Notably, we observed that these tumor cells penetrate the ovary and seed colonies within the ovarioles by physically displacing the egg chambers. Extending this analysis to the *pins* mutant tumors revealed that they, too, can metastasize to the ovary. However, *pins* tumors displayed a distinct invasion pattern, often filling the entire ovariole and encasing the egg chambers. These findings highlight how the nature of the initial genetic lesion can influence metastatic behavior and tissue colonization patterns. Importantly, we also demonstrate that epithelial tumors, previously unknown to invade the ovarian tissue, can invade through the ovarian tissue rather than grow as independent tumor masses. This expands the relevance of our model beyond NSC-derived tumors and establishes a robust system for studying the cell biology of tumor–host interactions.

We noticed significant thinning and deformation of host ovaries in all three tumor types analyzed. In many instances, these defects were observed often without any signs of invaded tumor cells (Fig. 5). Several studies, with genetic engineered tumors and allografted tumors, have indicated the involvement of host-derived secreted factors, resulting in ovarian tissue wasting (Hsi et al., 2023; Kim et al., 2021; Kwon et al., 2015; Liu et al., 2025). We surmised that these ovarian defects could be caused by cachexia induced by tumor-derived factors. However, how cachexia-induced tissue wasting could potentially influence metastatic invasion would be interesting to study.

We further extended our analysis to the *Drosophila* GI tract, a region that has received limited attention in the context of tumor metastasis, with existing studies on genetic models primarily noting tumor spread without detailed characterization (Bangi et al., 2012). Our findings reveal that tumors originating from both neural and epithelial tissues can associate closely with the outer surface of the GI tract, often spreading along its length and growing while physically attached. This external association led to visible constriction of the intestinal lumen; however, we did not observe tumor cell infiltration into the lumen itself. These results suggest that although the gut wall serves as a supportive substrate for tumor growth, it may possess intrinsic barriers that restrict deeper tissue invasion, in contrast to the ovary, where tumors readily penetrate multiple tissue layers. Additionally, we observed tumor association with the Malpighian tubules, although, similar to the GI tract, there was no evidence of tumor infiltration into these tissues. Together, these observations indicate that different host organs exhibit varying degrees of susceptibility to tumor invasion, likely governed by tissue-specific structural or molecular features. This underscores the value of the *Drosophila* model in uncovering how tissue architecture and microenvironment influence metastatic behavior.

In our study, we present a novel model to investigate tumor interactions with the adult *Drosophila* brain. We observed clear dissemination of NSC-derived tumors, originating from both *pins* and *lgl* mutants, to the adult brain (Fig. 7). Upon metastasis, these tumors exhibited a distinct pattern of growth, wrapping around the brain surface, and closely associating with and deforming the outer cortical layer of brain cells. Notably, despite extensive surface association and structural distortion of the cell cortex region, we did not observe tumor cell infiltration into the deeper neuropil region – where the majority of neuronal processes and synaptic connections reside. This spatial restriction suggests the presence of physical or molecular barriers that limit tumor penetration into neuronal tissue, or a lack of intrinsic invasive capacity in these tumor types to breach the brain parenchyma. These findings perhaps reflect the anatomical differences between *Drosophila* and mammalian brains; in *Drosophila*, the BBB encases the brain from the outside, which is an inside-out version of mammalian brains (Contreras et al., 2024; Limmer et al., 2014). In these models, *Drosophila* tumor cells interact with the BBB layer similarly to mammalian tumor brain metastasis and therefore provide an valuable tool to study cellular and molecular mechanism of brain tumor invasion.

### Conclusions

Despite its advantages, our *Drosophila* allograft model has certain limitations. Key among these is the challenge of distinguishing between passive tumor cell dissemination and active, invasive behavior – a distinction critical for understanding the mechanisms underlying true metastasis. T0 tumor cells originating from transplanted primary tissue and migrating to distant sites suggests that, following the tumor induction, tumor cells undergo active delamination from the primary tissue. However, whether the delaminated tumor cells actively migrate or passively disseminate to the distant sites is not known. However, we note that the active process of cell delamination could be less prominent in serially transplanted tumor cells, especially in the case of NSC-derived tumors. We notice that although tumor cells grow at the site of injection, in many cases, tumor clusters could also be seen at distant sites as early as Day 1-2 (Fig. 2), suggesting that tumor cells might enter the circulation at the time of injection without requiring any active delamination. Nevertheless, we provide evidence that these tumor cells do spread to the secondary host organs and show signs of secondary tumor colonies. So, although the allograft model might not be ideal for capturing some early steps of metastasis such as primary site exit, extravasation and intravasation, these models do successfully capture features of secondary metastasis sites, including tumor spread, invasion and secondary colony formation.

Additionally, although *Drosophila* provides a powerful genetic system, differences in tissue architecture and immune responses from those of vertebrates necessitate cautious interpretation when translating findings to human cancer biology. Nonetheless, this model offers unique strengths, including the ability to independently manipulate tumor and host genotypes, perform high-throughput genetic screens and visualize tumor–host interactions *in vivo* with temporal resolution. By integrating tumor-intrinsic properties with host tissue responses in a whole-organism context, our approach establishes a tractable and scalable platform for dissecting the complexity of metastatic progression. This work lays a foundation for uncovering conserved principles of cancer biology that will pave the way for future studies illuminating how tumors adapt, invade and colonize distant organ sites.

## MATERIALS AND METHODS

### *Drosophila* genetics and fly husbandry

Flies were raised on the standard Bloomington *Drosophila* Stock Center food recipe (LabExpress). Flies were cultured in an incubator maintained at 25°C and 40±5% relative humidity. No distinction was made between male and female larvae in the experiments conducted with larval tissue. Virgin female (3- to 4-day-old) flies were used as transplantation hosts.

#### *Drosophila* strains

*w^11-18^* (Bl. 3605); *lgl^4^-Frt40*, *Frt40* (Bl. 8212); *pins^62^*, *pins^83^* (Bl. 6497); *wor*-Gal4 (Bl. 56553); tub-Gal4 (Bl. 5138); *Frt40* MARCM, UAS-10X-IVS-mCD8::GFP (Bl. 32185 and (Bl. 32186); UAS-10X-IVS-mCD8::RFP (Bl. 32219); and Vkg::GFP (Bl. 98343) were from Bloomington *Drosophila* Stock Center.

### Transplantation of the primary tissue

Transplantation was carried out by adapting the protocol described previously (Rossi and Gonzalez, 2015). For microinjections, borosilicate glass capillary tubes (Narishige GD-1, Tritech Research) were pulled using a micropipette puller (Sutter Instruments, Model P-97). The capillary was attached to an aspirator connected to a mouth pipette (Tritech Research). Transplantation was performed using 3- to 5-day-old *w^11-18^* virgin female flies as the host. Primary tissues were dissected in *Drosophila* Schneider 2 (S2) cell culture medium from third-instar larvae. A specific injection protocol was followed for different tissues as detailed below.

#### *Drosophila* larval brain lobes

Third-instar larval brain lobes were dissected and separated into individual lobes (Fig. 1B1). Each intact brain lobe was aspirated in a microcapillary using mouth pipetting (Fig. 1B2,B3) and was implanted into the host abdomen by pipetting gently. Notably, aspiration of the brain lobe was easier to perform from the ventral nerve cord end. The first appearance of the T0 tumor was scored by visualizing GFP/RFP-positive small cell clusters ∼5-10 mm in size (Fig. 1B4, red arrow).

#### *Drosophila* larval imaginal discs

Third-instar larval wing discs were dissected in S2 cell medium (Fig. S4B1). Individual wing discs were aspirated in a microcapillary (Fig. S4B2,B3) for both control and tumorous genotypes and transferred to the host abdomen. The first appearance of the T0 tumor was scored by visualizing GFP/RFP-positive small cell clusters (Fig. S4B4, red arrow).

### Scoring of T0 tumor following transplantation of the primary tissue

Following the transplantation of the primary tissues into the host flies, the flies were allowed to rest overnight. The next day, the number of viable host flies was counted and recorded as the total number (*N*). Host flies were monitored daily for 30 days or until death to assess tumor growth (Fig. 1B4; Fig. S4A4, red arrow). Tumor-positive flies were transferred to a fresh vial and evaluated for additional parameters, such as secondary site metastasis.

### Serial transplantation of tumor mass

Flies harboring tumors were sorted under a fluorescence microscope, and those with significant tumor growth were selected. Tumor masses were isolated from the abdomens of host flies in sterile media under a fluorescence microscope, ensuring they were cleanly separated from host tissues. Following the isolation of the tumor mass, a specific protocol was employed to perform transplantations of tumors originating from different tumor types, as detailed below.

#### NSC-derived tumors

Tumor masses were isolated in sterile S2 cell culture medium from flies 8-10 days post-injection. The fly abdomen was opened in S2 medium to expose the tumor mass (Fig. 2A1), which was then separated from the abdomen using forceps and dissociated by aspirating in and out of a capillary to form tumor clusters (Fig. 2A2,A3). Approximately 100 nl of tumor cells were aspirated into the capillary and transplanted into the abdomens of 3- to 4-day-old female host flies (Fig. 2A4). Very fine capillary tips were used for secondary transplantation owing to their ease in aspirating tumor cells from primary tissues. This also provided the advantage of causing minimal damage to host flies, potentially reducing inflammatory responses.

#### Epithelial-derived tumors

Tumor masses were isolated in sterile S2 cell culture medium from flies 5-6 days post-injection. The fly abdomen was opened in S2 medium to expose the tumor mass, and the tumor mass was separated from the abdomen using forceps (Fig. S4D1). Instead of loosely attached tumor cell clusters, epithelial tumors grew as one to three solid masses. The tumor was dissociated into small pieces with the help of tweezers, followed by passing the pieces through a fine capillary (Fig. S4D2,D3). A small piece of tumor was aspirated into the capillary and transplanted into the abdomens of 3–4-day-old female host flies (Fig. S4D4). Owing to their intact and rigid structure compared to NSC-derived tumors, epithelial tumors were transplanted using a wider-bore capillary.

### Survival assay

3- to 4-day-old *w^11-18^* virgin female flies were injected with tumor cells at the desired transplantation stage. Control flies were injected with an equivalent amount of S2 medium. Following the injection procedure, the host flies were allowed to rest overnight, and the total number of viable flies was counted on Day 1, which was recorded as the total *N* for the experiment. After the injection, flies from both the tumor and media cohorts were monitored for viability once a day, and the surviving flies were counted. This procedure was continued until all flies in the tumor-injected cohort had died.

### Staining of the tumor mass

Host flies bearing tumors for 7-8 days were selected for staining the tumor mass. The flies were anesthetized, and their abdomens were carefully separated in 4% formaldehyde fixative to prevent spillage of the tumor mass. The abdomen was then opened using forceps to expose the tumor, taking care to avoid any tumor mass spillage. The dissected abdomens with exposed tumors were transferred to six-well glass plates and incubated in 4% formaldehyde for 20 min at room temperature. Following fixation, a standard tissue histochemistry protocol was performed as described below.

### Immunohistochemistry

A tissue-specific fixation and immunostaining protocol was followed. Dissected tissues were fixed in 4% formaldehyde and then incubated in blocking buffer containing 0.5% bovine serum albumin (BSA) in 0.1% PBST (1× PBS with 0.1% Triton X-100). Primary antibody incubation was carried out overnight at 4°C, followed by three washes in 0.1% PBST at room temperature. The following primary antibodies were used: rat anti-Dpn (11D1BC7; Abcam, ab195173, 1:100), rabbit anti-PH3 (Invitrogen, PA5-17869, 1:1000), mouse anti-Pros [Developmental Studies Hybridoma Bank (DSHB), MR1A, 1:25], mouse anti-Repo (DSHB, 8D12, 1:25) and rat anti-Elav (DSHB, 9F8A9, 1:10). After washing, tissues were incubated with Alexa Fluor 488-, 564- or 647-conjugated secondary antibodies (1:250) at room temperature, followed by three additional washes in 0.1% PBST. Following antibody staining, tissues were incubated with Phalloidin-Atto-647 (Sigma-Aldrich, 65906, 1:1000) for 15 min, then washed with 0.1% PBST. This was followed by incubation with an HSC NuclearMask (Invitrogen, H10325, 1:2000) for 10 min and a final wash with 0.1% PBST. Samples were then rinsed in 1× PBS and mounted on slides using Aqua-Poly/Mount (Polysciences, 18606-5). A detailed list of reagents is provided in Table S1.

### Imaging and image processing

Image acquisition was performed on a Zeiss laser scanning confocal microscope (Zeiss-880 and -980). For the whole organ (ovaries, GI tract and adult brain), titling imaging settings were used with 10% overlap between each tile. Image stitching was performed using Zen-blue software post-acquisition. The raw image data were processed using Fiji, and appropriate *z*-sections were maximum-intensity projected. Creative Cloud-Adobe Photoshop and Adobe Illustrator was used to adjust the intensity of the data and make the figures displayed in the paper.

### Statistical analysis

Data analysis was performed using Microsoft Excel and GraphPad Prism. All graphs show mean±s.e.m., with all individual data points displayed. A unpaired two-tailed Student’s *t*-test for the independent sample was performed for statistical significance. Data were determined significant if *P*<0.005.

## Supplementary Material

10.1242/dmm.052543_sup1 Supplementary information
