# Supplementary material for: Allograft transplantation for Drosophila tumor metastasis studies
Source: Dis Model Mech. 2025 Dec 29;18(12):dmm052543. doi: 10.1242/dmm.052543 (PMC12805642; doi:10.1242/dmm.052543)
Supplement: Supplementary information [file dmm-18-052543-s1.pdf]

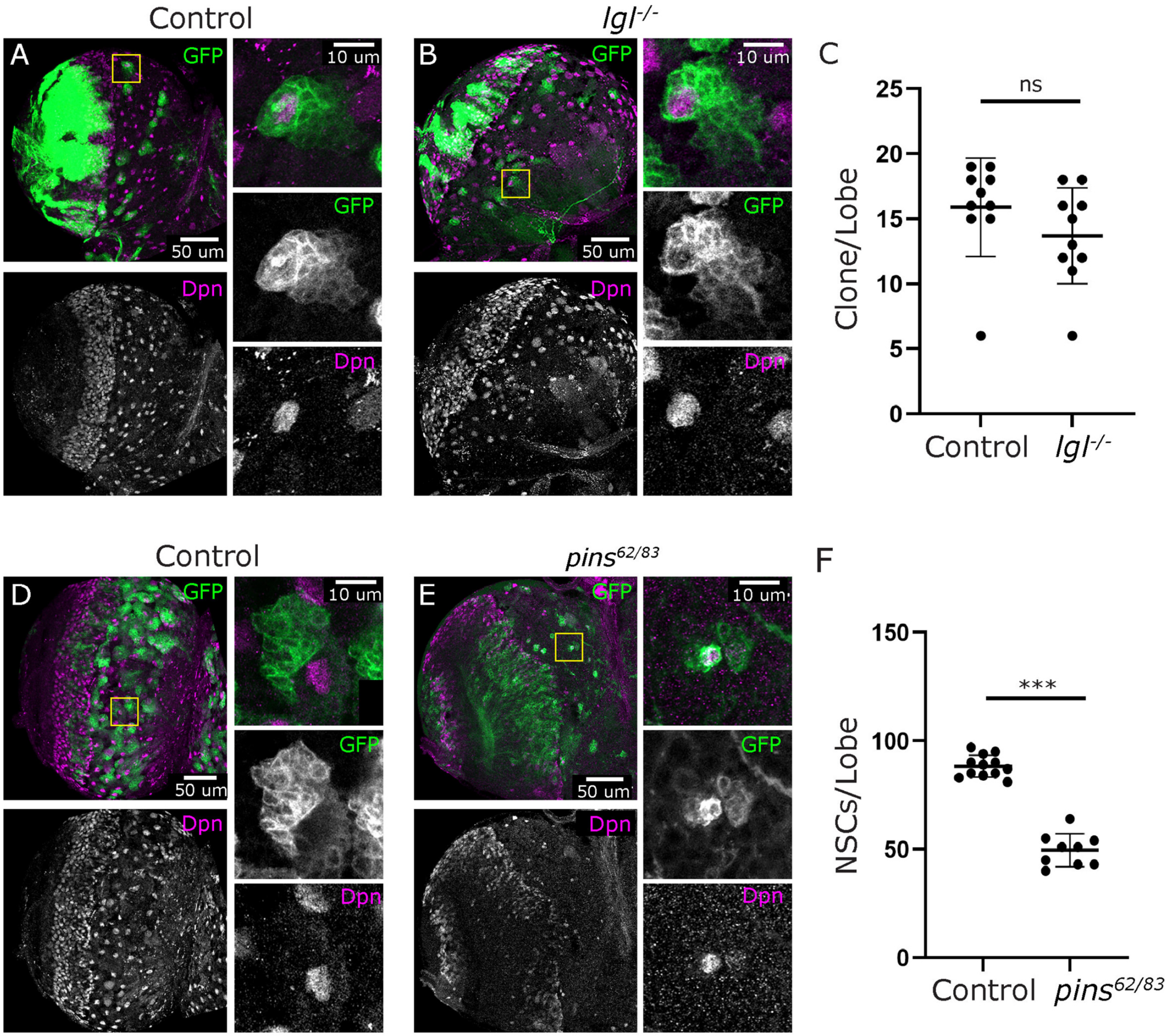

Disease Models & Mechanisms • Supplementary information

Fig. S1. Distinctive contribution of *lgl* and *pins* to NSCs proliferation in III instar larvae

(A-C) Third instar larval brain lobes harboring mCD8-GFP labeled MARCM clones (green), stained for deadpan (Dpn), the NSCs marker (magenta). (A) Control *Frt40* clones. (B) *lgl^{4}-Frt40* clones. (C) Number of NSCs clones per brain lobe (N=10 for A and N=10 for B). (D-F) Third instar larval brain lobes expressing mCD8-GFP under *wor-Gal4* (green), stained for deadpan (Dpn, magenta). (D) Control *wor-Gal4* clones. (E) *pins$^{62/83}$* clones. (F) Number of Dpn positive cells per brain lobe (N=12 for D and N=9 for E). Data is plotted as mean$\pm$ SEM, student t-test two-tailed for the independent sample, ns=not significant, ***=p<0.001. **Genotype:** (A) *hsflp; Gal80-FRT40/FRT40; tub-Gal4::UAS-mCD8-GFP*, (B) *hsflp; Gal80-FRT40/lgl^{4}-FRT40; tub-Gal4::UAS-mCD8-GFP*, (D) *wor-Gal4::UAS-mCD8-GFP*, and (E) *wor-Gal4::UAS-mCD8-GFP; pins^{62}/pins^{83}*

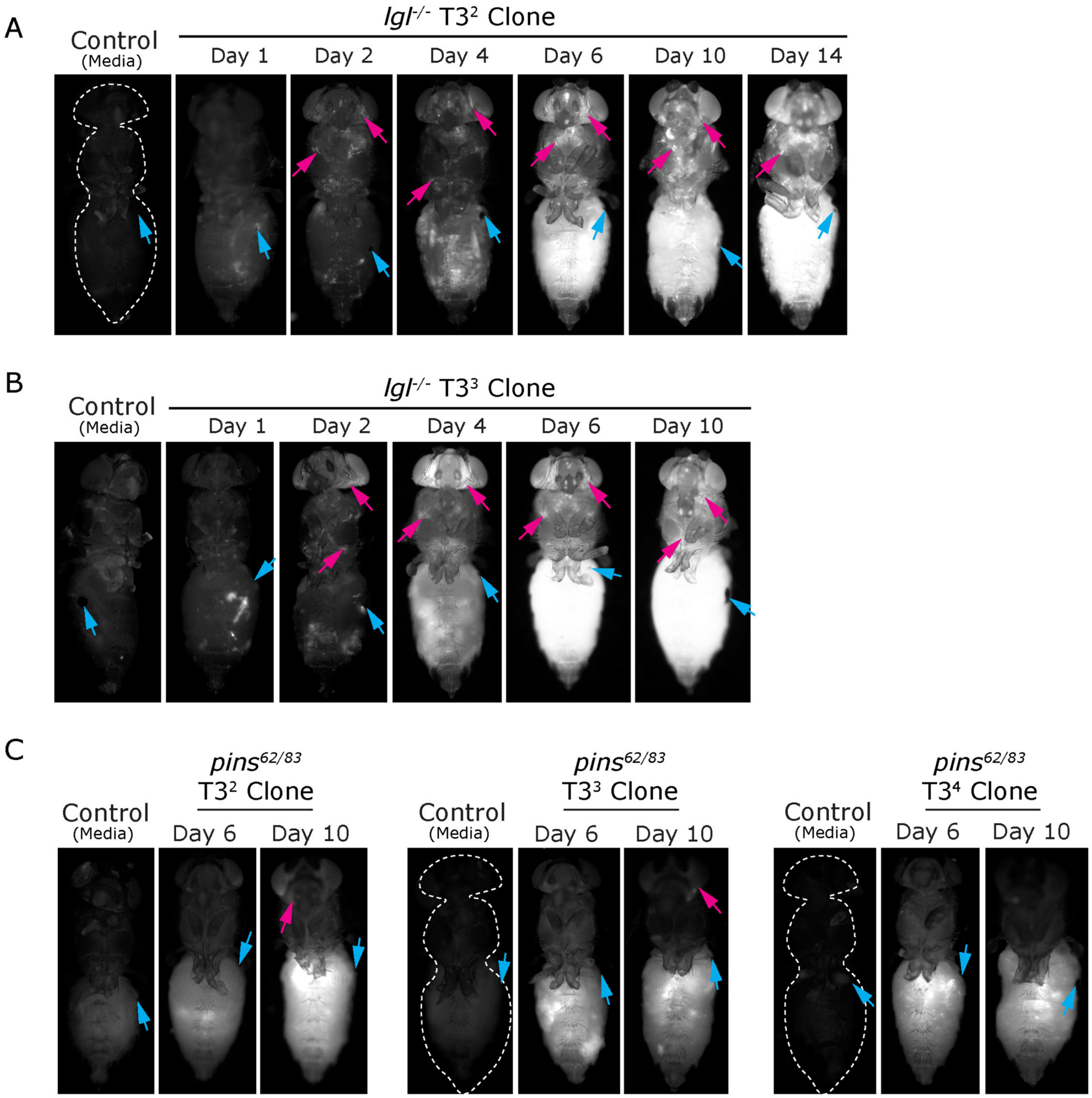

Fig. S2. Tumor progression of NSC-derived tumor

(A-B) Tumor progression of the *lgl*$^{-/-}$ T3 tumor at multiple days (Day 1- Day 10) following transplantation (Clone# 2 and 3). (C) Tumor progression of the *pins*$^{62/83}$ T3 tumor at Day 6 and Day 10 following tumor transplantation (Clone# 2, 3, and 4). (blue arrow = injection site, magenta arrows = tumor at distant sites). **Genotype:** (A-B) *w*$^{11\text{-}18}$ // media and *w*$^{11\text{-}18}$ // *lgl*$^{4/4}$-*mCD8-RFP* tumor (T3), and (C) *w*$^{11\text{-}18}$ // media and *w*$^{11\text{-}18}$ // *pins*$^{62/83}$-*mCD8-GFP* tumor (T3).

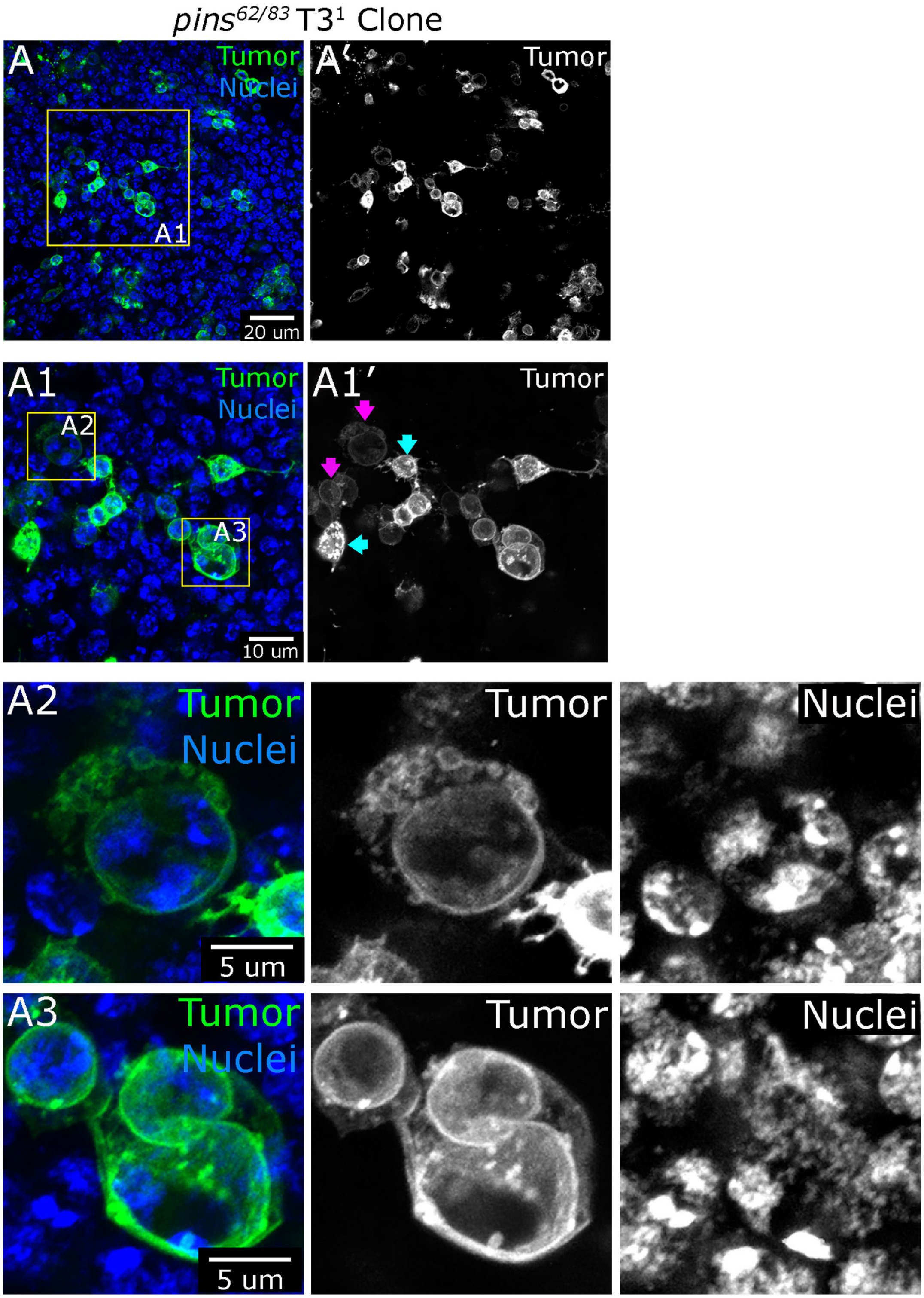

Fig. S3. The *pins* mutant tumor displays genomic instability

(A) *pins*$^{62/83}$ T3 tumor mass (green) stained with nuclear stain. (A1) Tumor cells expressing zero copy, one copy (magenta arrow), and <one copy (blue arrow) of mCD8-GFP. (A2-A3) Examples of multiple nuclei (membrane GFP, green and nuclear stain, blue). **Genotype:** (A, A1-A3) *w*$^{11\text{-}18}$ // *pins*$^{62/83}$*-mCD8-GFP* tumor (T3).

Disease Models & Mechanisms • Supplementary information

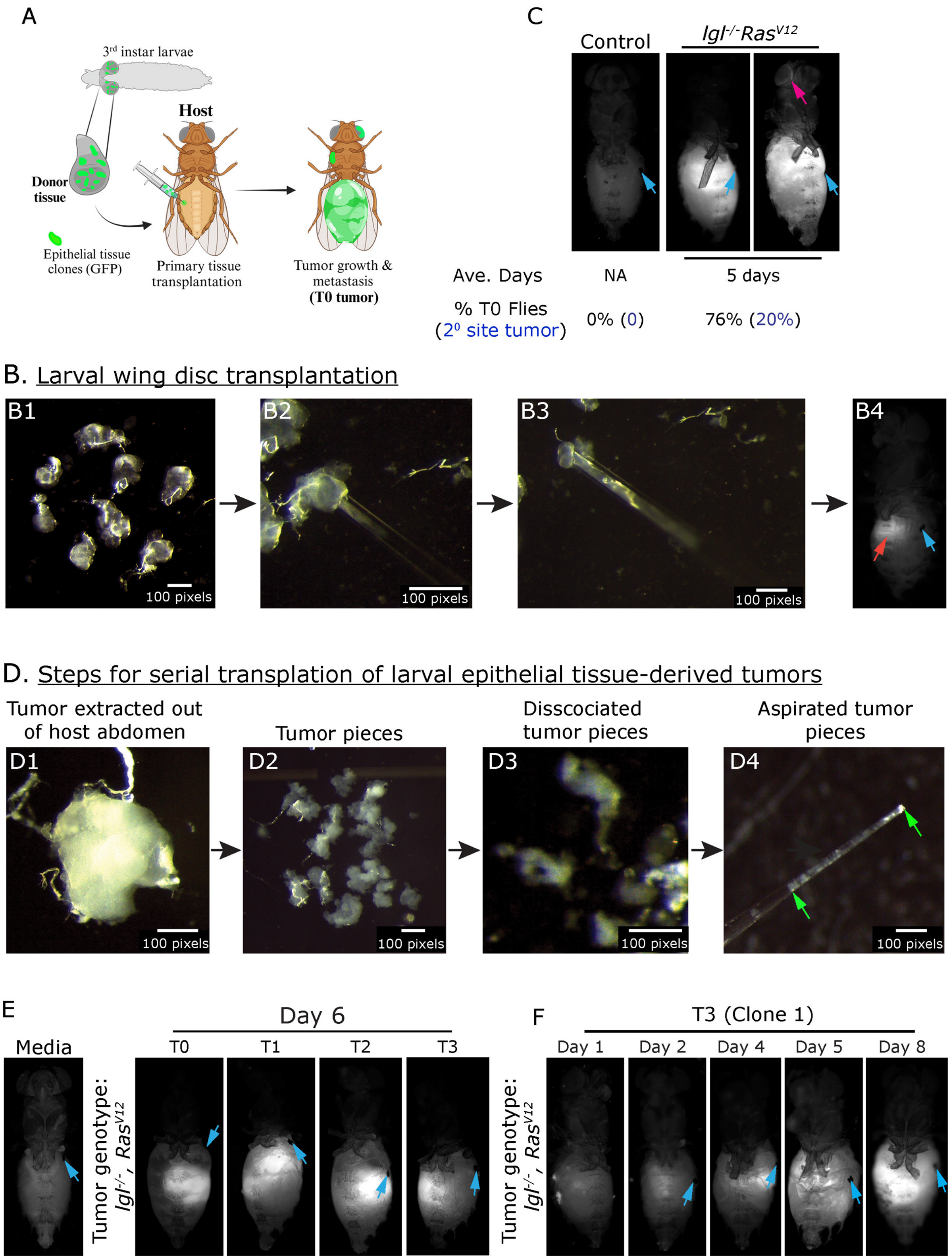

Disease Models & Mechanisms • Supplementary information

Fig. S4. Establishment, serial transplantation, and tumor progression of epithelial-derived tumors

(A) Schematic illustrating the allograft transplantation procedure using the III instar wing disc as donor tissue. (B) Bright-field images illustrating key steps of the wing disc transplantation procedure (see methods for details). (C) Fluorescence images of host flies taken 5 days after transplantation of wing discs from *Frt40* control (N= 15) and *lgl⁴-Frt40; Ras^{V12}* (N= 36). (D) Bright-field images illustrating key steps for performing serial transplantation of epithelial-derived tumors (see methods). (E) Host flies were at Day 6 through different stages (T0, T1, T2, T3) after serial transplantation of the *lgl$^{-/-}$ Ras^{V12}* tumor. Note the media injected control is reused from Fig. 4 as this is the same experiment with additional supplemental stages. (F) Tumor progression of *lgl$^{-/-}$ Ras^{V12}* T3 tumor at multiple days (Day 1- Day 8) following tumor transplantation. (yellow arrow= T0 tumor, blue arrow = site of injection, magenta arrow = tumor at distant sites, green arrow= aspirated tumor, red arrow= new T0 tumor). **Genotype:** (A) *w$^{11-18}$ // hsflp; Gal80-Frt40/Frt40; tub-Gal4::UAS-mCD8-RFP,* (B) *w$^{11-18}$ //hsflp; Gal80-Frt40/lgl^{4}-Frt40; tub-Gal4::UAS-mCD8-GFP/UAS-Ras^{V12}*, (C) *hsflp; Gal80-Frt40/Frt40; tub-Gal4::UAS-mCD8-GFP*, and *hsflp; Gal80-Frt40/lgl^{4}-Frt40; tub-Gal4::UAS-mCD8-GFP/UAS-Ras^{V12}*, (D) *w$^{11-18}$ // lgl$^{4/4}$; Ras^{V12}-mCD8-GFP* tumor (T0), and (E-F) *w$^{11-18}$* // and *w$^{11-18}$ // lgl$^{4/4}$; Ras^{V12}-mCD8-GFP* tumor (T3).

Disease Models & Mechanisms • Supplementary information

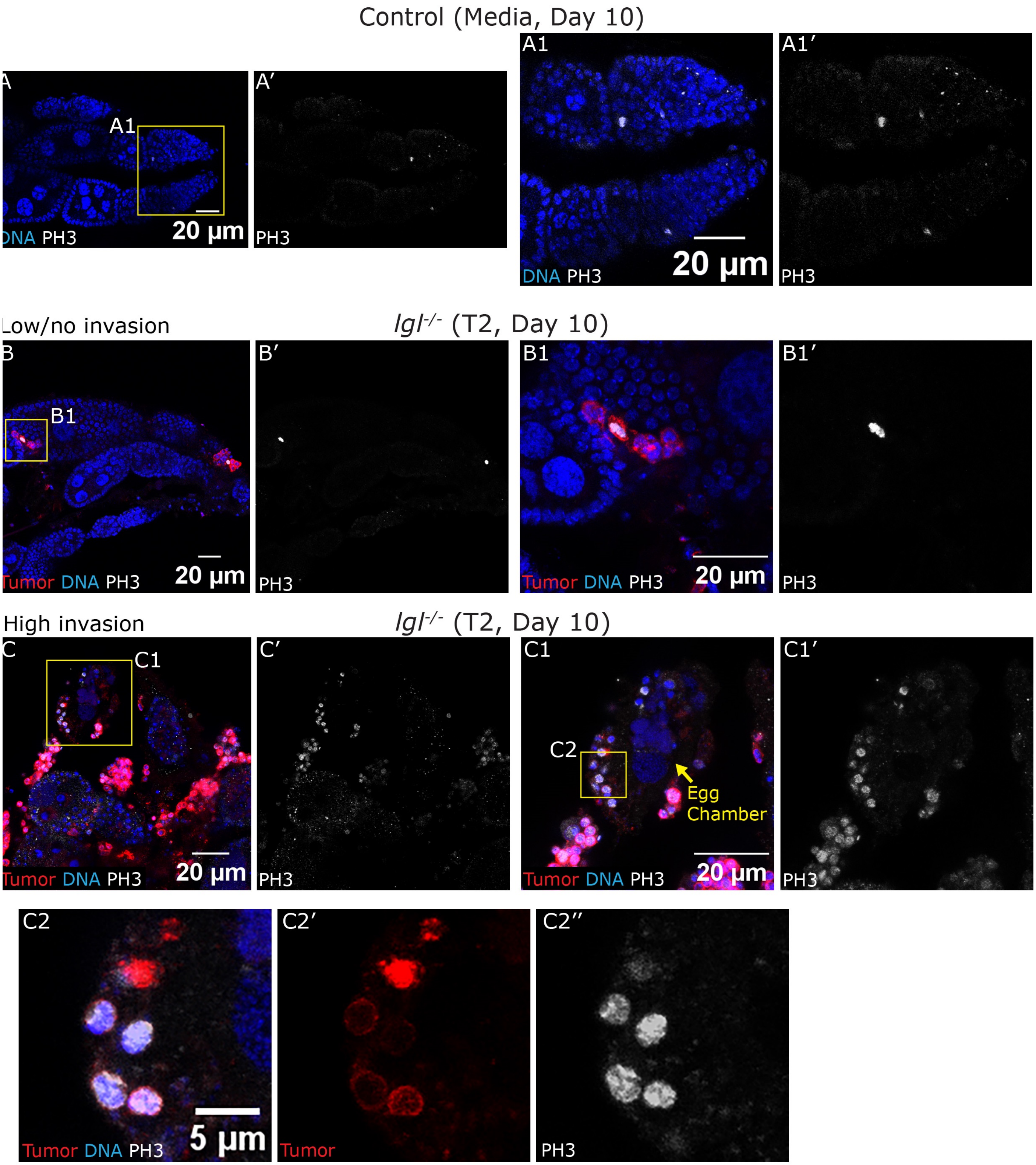
Control (Media, Day 10)
A
A1
20 µm
DNA PH3
A′
PH3
A1
DNA PH3
20 µm
A1′
PH3
Low/no invasion
lgl-/- (T2, Day 10)
B
B1
Tumor DNA PH3
20 µm
B′
PH3
B1
Tumor DNA PH3
20 µm
B1′
PH3
High invasion
lgl-/- (T2, Day 10)
C
C1
Tumor DNA PH3
20 µm
C′
PH3
C1
C2
Egg Chamber
Tumor DNA PH3
20 µm
C1′
PH3
C2
Tumor DNA PH3
5 µm
C2′
Tumor
C2′′
PH3

Fig. S5. Ovaries show signs of tumor proliferation at secondary site

(A) Ovary isolated 10 days after S2 media injection, stained with nuclear stain (blue). (B-B1) Host ovary (low invasion category) isolated 10 days after *lgl*$^{-/-}$ T2 tumors injection, showing tumor cells (GFP, red and nuclei, blue) and PH3 (grey, B'-B1'). (C-C1) Host ovary (high invasion category, egg chamber marked in yellow) isolated 10 days after *lgl*$^{-/-}$ T2 tumors injection, showing tumor cells (GFP, red and nuclei, blue) and PH3 (grey, C'-C1'). (C2) Blown-up image showing PH3 positive (C2'', Grey) tumor (red, C2') cells surrounding the egg chamber. **Genotype:** (A) $w^{11\text{-}18}$ // media, and (B) $w^{11\text{-}18}$ // *lgl*$^{4/4}$*-mCD8-GFP* tumor (T2).

Disease Models & Mechanisms • Supplementary information

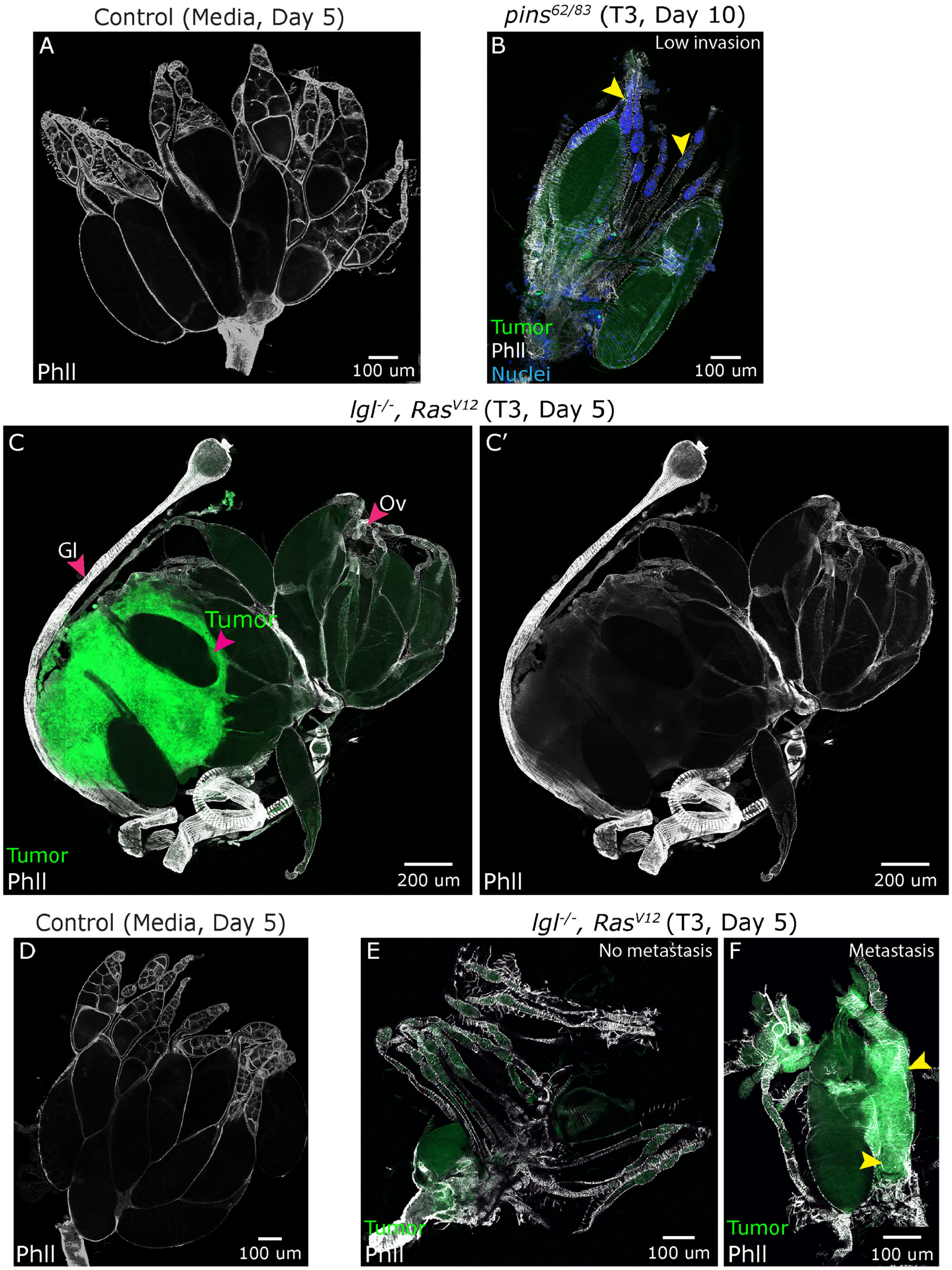

Disease Models & Mechanisms • Supplementary information

Fig. S6. Varying effects of tumor metastasis on the host's ovaries

(A) Ovary isolated 5 days after S2 media injection, stained with phalloidin (grey). (B) Host ovary (low invasion category) isolated 10 days after *pins*$^{62/83}$ T3 tumors injection, showing tumor cells (GFP, green and nuclei, blue), the yellow arrow points to ovarioles with tumor invasion. (C) *lgl*$^{-/-}$ *Ras*^{V12} tumor (green) along with the host fly's internal organs (Gastrointestinal (GI) tract, Ovaries (Ov)) stained with phalloidin (grey), isolated 5 days after T3 tumor injection. (D) Control ovary isolated 5 days after S2 media injection, stained with phalloidin (grey). (E-F) Ovary (phalloidin, grey) isolated 5 days after *lgl*$^{-/-}$ *Ras*^{V12} T3 tumor (green), showing examples with no metastasis (E) and metastasis (F) category ovaries, both categories displaying thinner and deformed ovaries. **Genotype:** (A) *w*$^{11-18}$ // media, (B) *w*$^{11-18}$ // *pins*$^{62/83}$*-mCD8-GFP* tumor (T3), (C) *w*$^{11-18}$ // *lgl*$^{4/4}$*; Ras*^{V12}*-mCD8-GFP* tumor (T3), (D) *w*$^{11-18}$, and (E-F) *w*$^{11-18}$ // *lgl*$^{4/4}$*; Ras*^{V12}*-mCD8-GFP* tumor (T3).

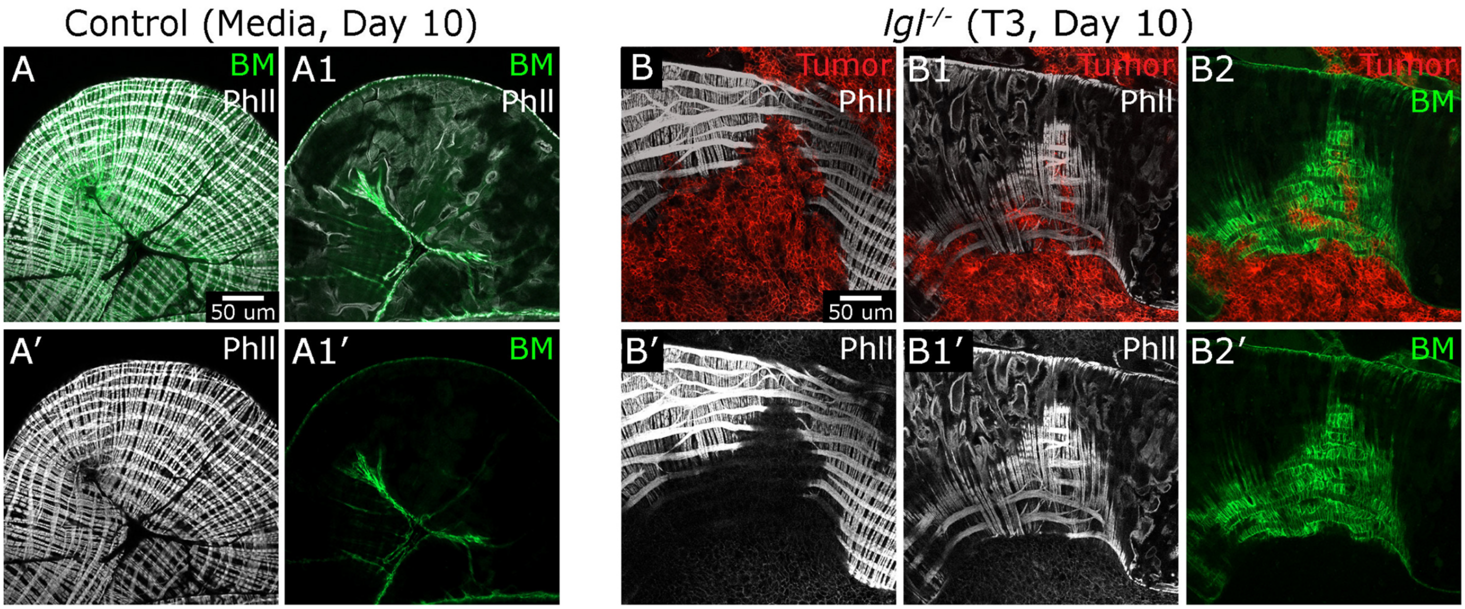

Fig. S7. *lgl* mutant tumor distort GI tract but do not infiltrate into gut lumen

(A–A1) Midgut region of the GI tract (corresponds to boxed region ‘a’ in Figure 6A) from control host flies 10 days after S2 media injection. (A) Surface Z-section showing basement membrane (BM, green) and visceral muscle labeled with phalloidin (grey).

(A1) Middle Z-section of the same midgut region showing internal organization of GI lumen.

(B–B2) Area-matched midgut region of the GI tract from *lgl*$^{-/-}$ tumor-injected host (corresponds to boxed region ‘b’ in Figure 6B). (B) Surface Z-section showing tumor cells (red) closely associated with the gut surface (visceral muscles, grey). (B1) Middle Z-section reveals displacement of visceral muscle (grey) by the tumor mass, with no tumor invasion into the gut lumen. (B2) Middle Z-section reveals displacement of GI wall with no apparent BM breach (green). **Genotype:** (A) *Vkg-GFP* // media, and (B) *Vkg-GFP* // *lgl*$^{4/4}$*-mCD8-RFP* tumor (T3)**.**

Disease Models & Mechanisms • Supplementary information

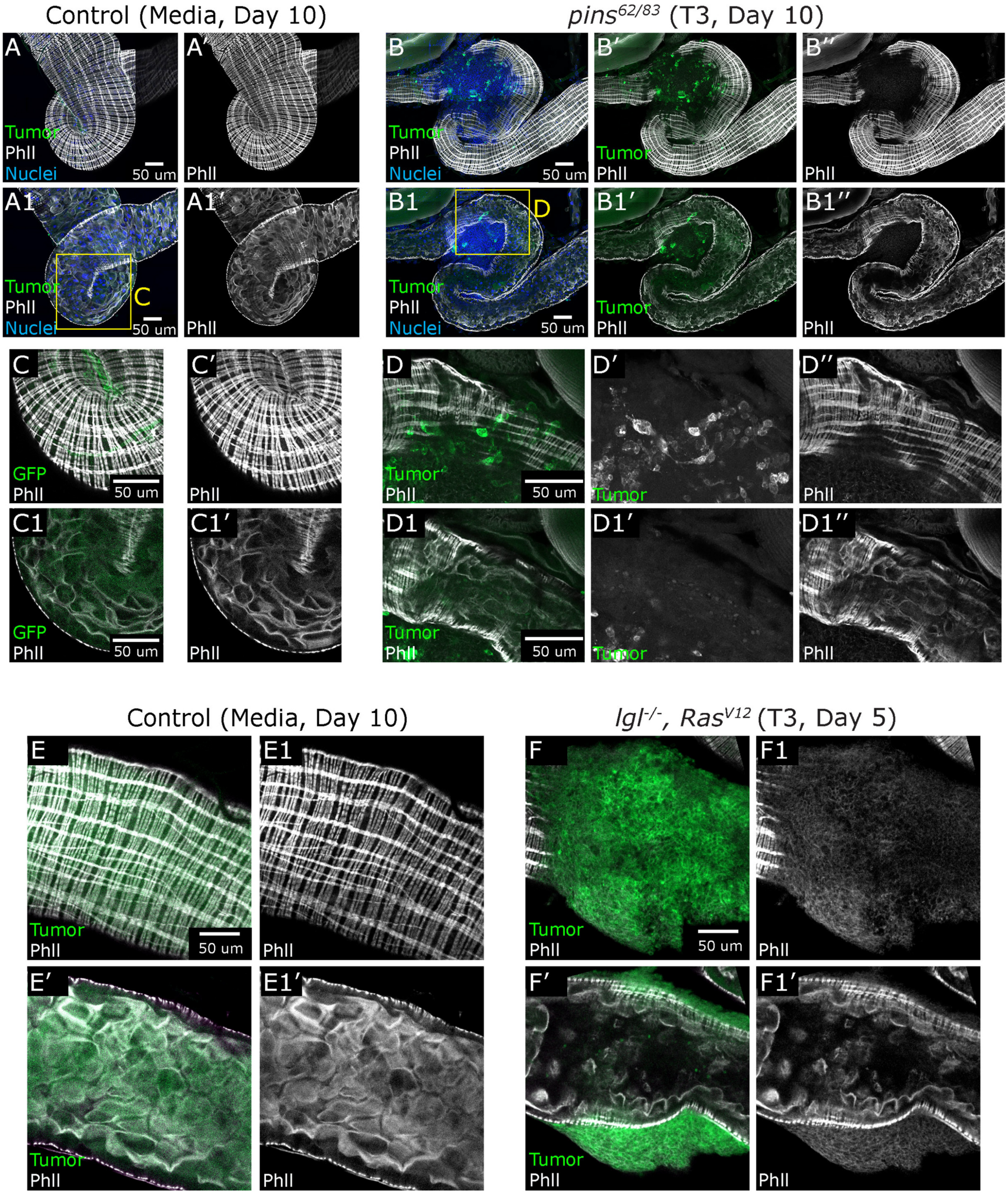

Disease Models & Mechanisms • Supplementary information

Fig. S8. NSC- and epithelial-derived tumors peripherally attach to GI without infiltrating the lumen

(A–A1) Midgut region of the GI tract from control host flies 10 days after S2 media injection. (A-A') Surface Z-section showing visceral muscle labeled with phalloidin (grey) and nuclei (blue). (A1-A') Middle Z-section of the same midgut region showing internal organization of GI lumen. (B–B2) Area-matched midgut region of the GI tract (corresponding to boxed region 'c' in Figure 6C) from *pins*$^{62/83}$ T3 tumors-injected host. (B-B'') Surface Z-section showing tumor cells (green) and tumor cell nuclei (blue) closely associated with the gut surface (visceral muscles, grey). (B1-B1'') Middle Z-section reveals displacement of visceral muscle (grey) by the tumor mass, with no tumor invasion into the gut lumen. (C) Blown-up images of box-C showing surface Z-section and middle Z-section of GI (GFP signal, green). (D-D1) Blown-up images of box-D showing surface Z-section and middle Z-section of GI, tumor (green), and visceral muscle (grey). (E-E1) Midgut region of the GI tract from control host flies 10 days after S2 media injection labeled with phalloidin (grey) and (GFP signal, green), (E-E') Surface Z-section and (E1-E1') Middle Z-section. (F-F1) Area-matched midgut region of the GI tract from *lgl*$^{-/-}$ *Ras*^{V12} T3 tumor-injected host (corresponds to boxed region 'd' in Figure 6D). (F) Surface Z-section showing tumor cells (green) decorating GI surface (visceral muscles, grey). (F1) Middle Z-section reveals displacement of visceral muscle (grey) by the tumor mass, with no tumor invasion into the gut lumen.

Genotype: (A and C) *w*$^{11\text{-}18}$ // media, (B and D) *w*$^{11\text{-}18}$ // *pins*$^{62/83}$*-mCD8-GFP* tumor (T3), (E) *w*$^{11\text{-}18}$ // media and (F) *w*$^{11\text{-}18}$ // *lgl*$^{4/4}$*; Ras*^{V12}*-mCD8-GFP* tumor (T3).

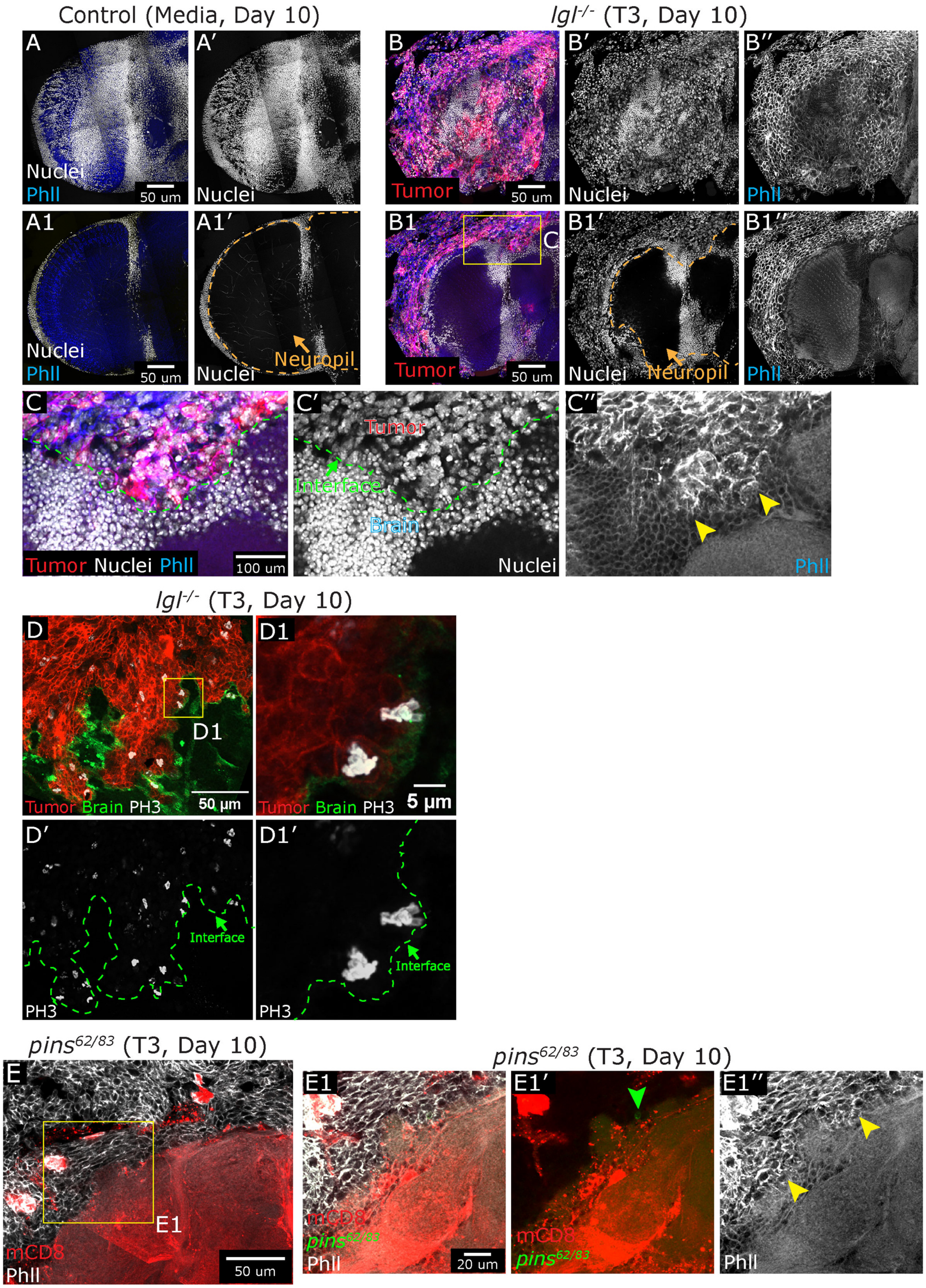

Control (Media, Day 10)
lgl-/- (T3, Day 10)
A
A′
B
B′
B″
Nuclei
PhII
50 um
Tumor
A1
A1′
B1
B1′
B1″
C
Neuropil
C′
C″
Interface
Brain
100 um
D
D1
D′
D1′
PH3
5 µm
50 µm
pins62/83 (T3, Day 10)
E
E1
E1′
E1″
mCD8
20 um

Fig. S9. *lgl* and *pins* mutant tumors to the adult fly brain

(A–A1) Optic lobe control host flies 10 days after S2 media injection (figure 7A, box "a"). (A-A') Surface Z-section showing cellular cortex marked with nuclei (grey). (A1-A1') Middle Z-section cellular cortex marked with nuclei (grey) and neuropil marked with phalloidin (blue and absence of nuclei, amber outline). (B-B1) Optic lobe isolated 10 days after *lgl*$^{-/-}$ T3 tumor injection (figure 7B, box "b"). (B-B'') Surface Z-section showing tumor cells (red) decorating the brain surface (marked by nuclei, grey). (B1-B1'') Middle Z-section showing cortical deformation by tumor and absence of tumor from neuropil region (amber outline and arrows). (C-C'') Blown-up area box-C marking tumor (red), brain (grey), and interface (green outline). Yellow arrows show tumor projections at the brain-tumor interface. (D-D') Surface section of tumor (red) decorating the brain (marked in green by perineural Gal4-GFP) showing actively phospho-histone 3 (PH3) positive tumor cells (grey). (D1-D1') Blown-up area box-D1 showing PH3 positive (grey) tumor cells (red) in close association with brain surface (green). (E-E1) Brain (red) injected with *pins*$^{62/83}$ T3 tumors, region from Figure 7D (box "e"). (E1-E1") Blown-up area box-D highlighting interface of tumor (grey) and brain (red). (E1') Tumor (grey plus rare GFP-positive green cell, green arrow). (E1") tumor projection at the brain-tumor interface (yellow arrow). **Genotype:** (A-D) *w*$^{11-18}$ // media, and *w*$^{11-18}$ // *lgl*$^{4/4}$*;* mCD8-RFP tumor and (E) *tub-Gal4::mCD8-RFP* // media and *tub-Gal4::mCD8-RFP* // *pins*$^{62/83}$-mCD8-GFP tumor (T3).

Disease Models & Mechanisms • Supplementary information

Table S1. Key Resources Table

Key Resources Table				
**Reagent type (species) or resource**	**Designation**	**Source or reference**	**Identifiers**	**Additional information**
gene (*D. melanogaster*)	*lgl* or *l(2)gl*			FLYBASE: FBgn0002121
gene (*D. melanogaster*)	*pins*			FLYBASE: FBgn0040080
gene (*D. melanogaster*)	Ras85D			FLYBASE: FBgn0003205
genetic reagent (*D. melanogaster*)	lgl^4-*Frt40*			FLYBASE: FBal0009225
genetic reagent (*D. melanogaster*)	*Frt40*		BDSC# 8212	FLYBASE: FBti0002071
genetic reagent (*D. melanogaster*)	$pins^{62}$			FLYBASE: FBal0104445
genetic reagent (*D. melanogaster*)	$pins^{83}$		BDSC# 6497	FLYBASE: FBal0117395

Disease Models & Mechanisms • Supplementary information

genetic reagent (*D. melanogaster*)	UAS-*Ras*^{V12}		BDSC# 64195	FLYBASE: FBti0012505
genetic reagent (*D. melanogaster*)	*wor-gal4*		BDSC# 56553	FLYBASE: FBti0161165
genetic reagent (*D. melanogaster*)	tub-Gal4		BDSC# 5138	FLYBASE: FBti0012687
genetic reagent (*D. melanogaster*)	*w*$^{11\text{-}18}$		BDSC# 3605	FLYBASE: FBal0018186
genetic reagent (*D. melanogaster*)	UAS-10X-IVS-mCD8::GFP		BDSC# 32185	FLYBASE: FBti0131931
genetic reagent (*D. melanogaster*)	UAS-10X-IVS-mCD8::GFP		BDSC# 32186	FLYBASE: FBti0131963
genetic reagent (*D. melanogaster*)	UAS-10X-IVS-mCD8::RFP		BDSC# 32218	FLYBASE: FBti0131950
genetic reagent (*D. melanogaster*)	VKG::GFP (GFP$^{vkg\text{-}G00454}$)		BDSC# 98343	FLYBASE: FBal0286156
Antibody	Mouse anti-Prospero (MR1A)	DSHB	RRID: AB_528440	1:50 dilution

Disease Models & Mechanisms • Supplementary information

Antibody	Mouse anti-Repo (8D12)	DSHB	RRID: AB_528448	1:50 dilution
Antibody	Rat anti-Elav (9F8A9)	DSHB	RRID: AB_528218	1:20 dilution
Antibody	Rat anti-Deadpan (11D1BC7)	ABCAM-195173	RRID: AB_2687586	1:100 dilution
Antibody	Rabbit anti-PH3	Invitrogen PA5-17869	RRID: AB_10984484	1:1000 dilution
Nuclear Stain	HCS NuclearMask Stains	Invitrogen H10325		1:2000 dilution
Phalloidin	Phalloidin–Atto 647N	Sigma 65906		1:1000 dilution
Mounting medium	Aqua-Poly/Mount	Polysciences 18606-5		
Culture medium	*Drosophila* S2 Cells	Gibco 21720024		
